# Ecteinascidin synthetic analogues: a new class of selective inhibitors of transcription, exerting immunogenic cell death in refractory malignant pleural mesothelioma

**DOI:** 10.1186/s13046-024-03253-y

**Published:** 2024-12-21

**Authors:** I. C. Salaroglio, P. Aviles, J. Kopecka, A. Merlini, F. Napoli, L. Righi, S. Novello, H. Sullivan, C. Cuevas, G. V. Scagliotti, C. Riganti

**Affiliations:** 1 Department of Oncology, Molecular Biotechnology Center “G. Tarone”, University of Torino, Piazza Nizza 44, Torino, 10126 Italy; 2 PharmaMar S.A, Avda de los Reyes 1, Colmenar Viejo, Madrid, 28770 Spain; 3 Department of Oncology at San Luigi Gonzaga Hospital, Medical Oncology Unit, University of Torino, Regione Gonzole 10, Orbassano, 10043 Italy; 4 Department of Oncology at San Luigi Gonzaga Hospital, Pathology Unit, University of Torino, Regione Gonzole 10, Orbassano, 10043 Italy

**Keywords:** Malignant pleural mesothelioma, Ecteinascidins, DNA damage, Immunogenic cell death, cGAS/STING pathway, Chemo-immunotherapy

## Abstract

**Background:**

Malignant pleural mesothelioma (MPM) is a highly chemo-refractory and immune-evasive tumor that presents a median overall survival of 12–14 months when treated with chemotherapy and immunotherapy. New anti-tumor therapies as well as the concomitant reactivation of immune destruction are urgently needed to treat patients with this tumor. The aim of this work is to investigate the potential effect of ecteinascidin derivatives as lurbinectedin as new first-line treatment option in MPM, alone and in combination with immunotherapy.

**Methods:**

The antitumor activity of ecteinascidin synthetic analogues: lurbinectedin, ecubectedin and PM54 was evaluated in an array of patient-derived MPM cells in terms of cell proliferation, cell cycle, apoptosis, DNA damage and repair. Immunoblot was used to assess the cGAS/STING pathway. ELISA and flow cytometry-based assays were used to evaluate immunogenic cell death parameters and the effect on the immunophenotype in autologous peripheral blood monocyte-MPM cells co-cultures. Patient-derived xenografts (PDX) in humanized mice were used to evaluate the efficacy of ecteinascidins in vivo.

**Results:**

Lurbinectedin, ecubectedin, and PM54 were effective in reducing cell proliferation and migration, as well as inducing S-phase cell cycle arrest and DNA damage in malignant pleural mesothelioma cells. These effects were more pronounced compared to the standard first-line treatment (platinum-based plus pemetrexed). Mechanistically, the drugs downregulated DNA repair genes, activated the cGAS/STING pathway, and promoted the release of pro-inflammatory cytokines. They also induced immunogenic cell death of mesothelioma cells, enhancing the activation of anti-tumor CD8^+^T-cells and natural killer cells while reducing tumor-tolerant T-regulatory cells and myeloid-derived suppressor cells in ex vivo co-cultures. These promising results were also observed in humanized patient-derived xenograft models, where the drugs were effective in reducing tumor growth and increasing the ratio anti-tumor/pro-tumor infiltrating immune populations, either alone or combined with the anti-PD-1L atezolizumab.

**Conclusions:**

Collectively, these findings reveal a previously unknown mechanism of action of ecteinascidins that merits further investigation for potential clinical applications in the treatment of MPM, as new first line treatment in monotherapy or in association with immunotherapy.

**Supplementary Information:**

The online version contains supplementary material available at 10.1186/s13046-024-03253-y.

## Introduction

Malignant pleural mesothelioma (MPM), a highly aggressive tumor primarily linked to exposure to asbestos often presents at an advanced stage and has a long latency period, sometimes exceeding 30 years, making diagnosis challenging [1]. MPM is categorized into three histotypes: epithelioid (50–60% of cases), which typically has a more favorable prognosis; sarcomatoid (10% of cases), which is drug-resistant and associated with a poorer prognosis; and biphasic (30–40% of cases), where the tumor contains varying proportions of the other two histotypes [1]. Despite these distinctions, available treatments are limited and offer modest clinical benefits, resulting in overall survival (OS) rates ranging from 12 to 36 months. The recommended initial treatment for MPM is a combination of platinum-based chemotherapy with pemetrexed (Pt + PMX), either with or without the addition of bevacizumab [2, 3]. The microenvironment of MPM is highly infiltrated by immunosuppressive cells which justifies the exploratory evaluation of treatments based on immune checkpoint inhibitors (ICIs) nivolumab plus ipilimumab [4–6]. The phase III open label study Checkmate 743, which compared the chemo-free doublet of nivolumab and ipilimumab versus Pt + PMX, in 605 PD-L1 unselected patients showed that OS was significantly improved in the experimental arm (18.1 versus 14.1 months) but only in non-epithelioid subtypes [7]. OS is better with chemotherapy in epithelioid MPM and with ICIs in sarcomatoid MPM patients. Although none of this first line (1 L) treatment produced improvements [6], additional phase III studies of combined chemo-immunotherapy are largely awaited [7]. Moreover, despite efforts made with different therapeutic approaches, the approval of a second-line (2 L) therapy is still pending [2–8].

The lack of an effective targeted therapy may be explained through the genomic heterogeneity of MPM. Most frequent mutations in MPM, i.e., *BRCA1-associated protein* (*BAP1)*, *CDKN2A*,* NF2*, *TP53* and *SETD2*, are tumor suppressor inactivating genes [9]. Mutations in BAP1, detected in 30-60% of MPM cases, accelerate asbestos-induced MPM in mice [1]. BAP1 is an oncosuppressor gene regulating chromatin remodeling, DNA replication under stress conditions and DNA damage response, by recruiting RAD51 to the locus of damaged DNA. Thanks to these pleiotropic functions, BAP1 controls cell cycle progression, apoptosis and differentiation [10].

Lurbinectedin is a synthetic compound that preferentially binds to CG-rich sequences in the minor grove of DNA near gene promoters, inhibiting transcription [11–13]. Lurbinectedin affects the viability of monocytes and tumor associated macrophages (TAMs), inhibits the secretion of immunosuppressive and proangiogenic cytokines *CCL2*, *CXCL8* and *VEGF* by monocytes [14]. Clinical trials in solid tumors have demonstrated effective antitumor activity with manageable side effects such as temporary grade 4 neutropenia, fatigue, and vomiting at the recommended dose [15–19]. After promising results of a second line (2 L) clinical trial [20], lurbinectedin has been approved in sixteen different countries, worthy of mention, United States (https://www.fda.gov/) and Canada (https://www.canada.ca/) to treat metastatic small cell lung cancer (SCLC) with disease progression during or post platinum-based chemotherapy. However, the phase 3 ATLANTIS study on relapsed SCLC, indicated a better safety profile of lurbinectedin combined with doxorubicin versus chemotherapy alone, despite no significant differences in OS [21]. Additionally, recent results of the Phase 3 IMforte trial have shown a statistically significant benefit for lurbinectedin and atezolizumab combination in extensive-stage SCLC patients receiving this treatment in a 1 L maintenance setting [21]. On the other hand, the other two synthetic lurbinectedin analogs, ecubectedin and PM54 (CT-EU-00057825), are currently undergoing the initial stages of clinical development (phase 1 or phase 2 studies) [22, 23].

In both SCLC and MPM, lurbinectedin used as at least third line (3 L) treatment, did not show benefit in OS but the drug displayed immunomodulatory functions, as the increase in natural killer (NK) cells, and the change in the pattern of co-stimulatory/co-inhibitory receptor on CD4^+^ and CD8^+^ circulating T-lymphocytes [24]. Similarly, the SAKK17/16 study on 42 MPM patients where 1 L and 2 L treatment had failed, demonstrated that lurbinectedin slightly increased OS in patients with low intratumoral T-regulatory (Treg) cells and TAMs, exerting a positive effect on MPM microenvironment (i.e., a decrease in M2-polarized macrophages and an increase in infiltrating CD8^+^T-cells) [25]. Collectively, these results set the rational basis for the potential use of the drug in combination with ICIs, given the drug’s immunomodulatory properties. The mechanism underlying immune-activation and the benefit of chemo-immunotherapy based on ICIs, however, have not been investigated.

In this work, the preclinical activity of three ecteinascidin synthetic compounds, namely lurbinectedin, ecubectedin and PM54, has been proven on a panel of primary MPM cultures and in two patient-derived xenografts (PDXs) implanted in mice with a human reconstituted immune system. Ecubectedin and PM54 showed the best antitumor activity, particularly when combined with anti-PD-1 atezolizumab. The main mechanisms of anti-tumor activity relies on the drugs’ ability to exert a strong DNA damage and increase MPM cells immunogenicity, activating STING pathway and reshaping MPM immune-environment in an antitumor manner.

## Materials and methods

### Reagent and chemicals

Plasticware was obtained from Falcon (Glendale, AZ, USA). Electrophoresis reagents were acquired from Bio-Rad Laboratories (Hercules, CA, USA). Unless specified otherwise, all other reagents were purchased from Sigma Chemicals Co. (St. Louis, MO, USA).

### Cells

Primary MPM cells were obtained from pleurectomy or explorative thoracoscopy performed at AOU Città della Salute e della Scienza (Torino), AOU San Luigi Gonzaga (Orbassano) and AO of Alessandria (Italy), after written informed consent. The study was approved by the local Ethical Committee (#128/2016). Supplementary Table S1-S2 summarize clinical and histological characteristics. Anonymized MPM samples were used until cell passage 5 as 2D-cultures or spheroids [26], monitored by contrast phase microscopy (Leica DC100 microscope, Leica Microsystems GmbH, Wetzlar, Germany).

The pathological characterization was performed by immuno-histochemistry analyses using the following antibodies: calretinin (RB-9002-R7; Thermo Fisher Scientific, Waltham, MA, USA), pancytokeratin (clone AE1/AE3; Dako, Santa Clara, CA, USA), podoplanin (clone D2-40; Dako), epithelial membrane antigen (EMA, clone E29; Dako), carcino-embrionic antigen (CEA, IR52661-2; Dako), Wilms tumor-1 antigen (WT1, clone 6FH2: Thermo Fisher Scientific), cytokeratin 5 (clone D5; Menarini Diagnostics, Bagno a Ripoli, Italy) using an automated immunostainer (Benchmark Ventana Medical Systems, Tucson, AZ, USA). Samples were diagnosed as MPM if positive for at least one mesothelial antigen among calretinin, WT1, podoplanin and cytokeratin 5, or positive for pancytokeratin. Cells were cultured in HAM F12/DMEM medium supplemented with 1% penicillin-streptomycin (PS) and 10% fetal bovine serum (FBS). In MPM 3D cultures, 1 × 10^5^ cells were seeded in HAM F12/DMEM medium supplemented with 1% PS, 20 ng/ml of EGF, 20 ng/ml of β-FGF, 4 µg/ml of IGF, 0.2% v/v B27 [26], and grown for 6 weeks to obtain MPM spheroids. Cells and spheroids were monitored by a contrast phase Leica DC100 microscope.

### In vitro antitumor activity

Antitumor activity was assessed by crystal violet staining [27]. In short-term viability assays, 2 × 10^3^/well were seeded into 96-well plates and incubated for 72 h with serially diluted (0.01 nM–100 nM) lurbinectedin, ecubectedin, PM54 and Pt + PMX, as first-line treatment (0.1 nM–100 nM). Cell viability was measured by crystal violet assay through which cells were fixed and stained with 5% w/v crystal violet solution in 66% w/v methanol, and washed with sterile water. Crystal violet was eluted by adding 10% v/v acetic acid into each well. Quantification was performed by measuring the absorbance (570 nm) with Cytation 3 Imaging Reader (Bio-Tek Instruments, Winooski, VT, USA). IC_50_ and IC_10_ were calculated with GraphPad PRISM software (v.9.4.1). In long-term viability assays, cells were seeded at a density of 4 × 10^3^/well in 12-well plates and treated with the concentrations of lurbinectedin, PM14, PM54 and Pt + PMX corresponding to IC_10_ for 10 weeks (4 weeks: one treatment/week; 2 weeks: drug holiday; 4 weeks: one treatment/week). Cell viability was measured by crystal violet staining. 3D-cultures were treated with lurbinectedin, ecubectedin, PM54 and Pt + PMX at IC_50_ of 2D-cultures for 72 h and monitored by a contrast phase Leica DC100 microscope from Leica Microsystems GmbH, Wetzlar, Germany.

### Migration and invasion assays

MPM cells were scratched (at a density of 90–100% confluence in 6-well plates) using a 200-µl sterile tip, washed twice with PBS, and treated with lurbinectedin, ecubectedin, PM54 and Pt + PMX, at IC_50_ concentration. Migration of cells was observed under monitored by a contrast phase Leica DC100 microscope at t0, after 24 h and 48 h. The percentage of migrated cells (i.e., number of cells counted within the scratch/number of cells seeded) was calculated using ImageJ software. For invasion assay [28], MPM cells, re-suspended in 0.45% type VII low-melting agarose that contained medium supplemented with 10% FBS at 1 × 10^5^ cells/well, were plated in the upper chamber of 6-well Transwell plates on a layer of 0.9% agarose, diluted in complete medium, and cultured for 3 weeks with the concentrations of lurbinectedin, ecubectedin, PM54 and Pt + PMX that corresponded to the IC_10_, once/weekly. After this, cells that migrated to the lower chamber were stained by crystal violet. Images of the lower chamber were acquired by a contrast phase Leica DC100 microscope. To obtain the percentage of migrated cells/field (number of cells in the lower chamber or number of cells seeded in the upper chamber), quantification was performed by measuring absorbance with the Cytation 3 Imaging Reader (Bio-Tek Instruments).

### Cell cycle analysis and apoptosis

Cells were plated in 6-well plates -at a density of 1.2 × 10^5^/well-, and treated with lurbinectedin, ecubectedin, PM54 and Pt + PMX at IC_5__0_ for 24 h. Subsequently, cells were washed with PBS, treated with RNAse (167 µg/mL), and stained for 15 min at RT with propidium iodide (PI; 33 µg/mL). The cell-cycle distribution in G0/G1, S, and G2/M phases was analyzed by FACSCalibur flow cytometer (Becton Dickinson, Franklin Lanes, NJ, USA) and calculated using the CellQuest program (Becton Dickinson). MPM cells were plated in 6-well plates -at a density of 1.2 × 10^5^/well-, and treated with lurbinectedin ecubectedin, PM54 and Pt + PMX at IC_50_ concentration for 24 h. Floating and adherent cells were washed with PBS and stained with the Annexin V-FITC Apoptosis Detection Kit (Sigma). The percentage of necro-apoptotic (Annexin V FITC^+^/PI^+^) cells was measured by FACSCalibur flow cytometer and calculated using the CellQuest program.

### PCR arrays

PCR arrays were carried out on 1 µg cDNA, obtained with iScriptTM cDNA Synthesis Kit (Bio-Rad Laboratories), using the DNA-damage-induced responses RT2 Profiler PCR Array (Bio-Rad Laboratories) as per Manufacturer’s instructions. Data analysis was performed using PrimePCR™ Analysis Software (Bio-Rad Laboratories).

### Comet assay

DNA damage was assessed by Single Cell Gel Electrophoresis assay (Comet assay; [29]). A minimum of 100 nuclei were counted in each condition and the percentage of DNA in the tail was quantified with CometScore software (TriTek Corp., Sumerduck, VA, USA).

### Immunoblotting

Cells were incubated on ice for 20 min in 0.1% of Triton X-100 lysis buffer (20 mM Tris HCl pH 7.4; 150 mM NaCl; 5 mM EDTA; 0.1% Triton X-100; 1 mM phenylmethanesulfonyl fluoride; 10 mM NaF; 1 mM Na_3_VO_4_, supplemented with protease inhibitor cocktail III, Merck), sonicated and centrifuged at 13,000×rpm for 10 min. 30 µg of protein lysates, resolved by 4–15% Mini- PROTEAN TGX Precasted Protein Gels (Bio-Rad Laboratories), were blotted with the following antibodies: cGAS (79978, Cell Signalling, Danvers, MA, USA); STING (13647, Cell Signaling); p-TBK1 (PA5-105919, Invitrogen, Milano, Italy); TBK1 (PA5-17478, Invitrogen); p-IRF3 (37829, Cell Signalling); IRF3 (4302, Cell Signalling); p-IKKα/β (2697, Cell Signalling); IKKα/β (SN63-02, Invitrogen); Actin (5174, Cell Signalling), followed by horseradish peroxidase-conjugated secondary antibodies (Bio-Rad Laboratories). Proteins were detected by enhanced chemiluminescence kit (Bio-Rad Laboratories). The band intensity was calculated with the Image J software, as ratio between density of protein of interest/density of housekeeping protein, considered 100%, and – for TBK1, IRF3, IKKα/β – as ratio between mean of phosphorylated/total proteins.

### NF-kB activation

10 µg of nuclear proteins, extracted with the Nuclear Extract Kit (Active Motif, La Hulpe, Belgium), were used to measure NF-kB nuclear translocation and DNA binding using the TransAM^®^ NF-kB Activation Assay Kit (Active Motif).

### Cytokines detection

The following cytokines were quantified in MPM culture supernatants using specific kits: IFN-γ (IFN-γ DuoSet Development Kit (R&D Systems, Minneapolis, MN, USA), IFN-β (VeriKine Human IFN Beta ELISA Kit, PBL Assay Science, Piscataway, NJ, USA), CXCL5 (ENA-78, Invitrogen), CXCL10 (KAC236, Invitrogen), TNF-α (KHC3011, Invitrogen), IL-6 (KHC0061, Invitrogen), and IL-12 (KAC1568, Invitrogen).

### In vitro assessment of immunogenic cell death induction

Three markers of in vitro immunogenic cell death (ICD) – surface calreticulin (CRT), extracellular ATP and high-mobility group box-1 (HMGB1) protein − [30] were quantified as described [31, 32]. Surface calreticulin was measured by flow cytometry [31]. The amount of released ATP was measured with ATP Determination Kit (A22066, Invitrogen), using a Cytation 3 Imaging Reader (Bio-Tek Instruments). ATP was quantified as arbitrary light units; data were converted into nmoles/mg proteins. The extracellular release of the HMGB1 was measured with the High Mobility Group Box 1 ELISA kit (ABIN6574155, Antibodies-Online, Aachen, Germany). Results were expressed in pg/mg total cellular proteins.

### Phagocytosis, T-lymphocyte activation and immune-killing

Dendritic cells (DCs) were generated by healthy donors from Blood Bank of AOU Città della Salute e della Scienza (Torino, Italy) [31]. Phagocytosis assays were performed as detailed [31]. Active anti-tumor cytotoxic CD8^+^CD107a^+^INFγ^+^T-lymphocytes were obtained from autologous T-lymphocytes co-cultured with DC and quantified by flow cytometry [33]. MPM cells were collected, co-cultured with T-lymphocytes, and stained with the Annexin V-FITC Apoptosis Detection Kit (Merck) to detect necro-apoptotic cells, as index of immune-killing [33].

### Immune-phenotyping, immune checkpoints and immune-senescence markers

Peripheral blood mononuclear cells (PBMC) were incubated for 5 days with MPM cells [31], harvested, re-suspended in PBS containing 5% FBS and stained with the appropriate combination of antibodies (all from Miltenyi Biotec., Bergish Gladbach, Germany; diluted 1:10): CD3 (REA613), CD4 (M-T466), CD8 (BW135/80) for T-lymphocytes; CD56 (AF127H3), CD335/ NKp46 (9E2) for NK cells; CD4 (M-T466), CD25 (4E3), CD127 (MB1518C9) for Treg cells; CD14 (TUK4) and CD68 (Y1/82A) for monocytes and macrophages; CD11b (M1/ 70.15.11.5), CD14 (TUK4), CD15 (VIMC6), HLADR (AC122) for granulocyte-derived myeloid derived suppressor cells (Gr-MSDC) and monocyte-derived myeloid derived suppressor cells (Mo-MDSC).

For immune checkpoints (ICPs) and their ligands, and immunosenescence markers, PBMC co-cultured with MPM cells were collected. T-lymphocytes, isolated with the Pan T Cell Isolation Kit (Miltenyi Biotec.), were stained with the following antibodies: CD277/HVEM (HMHV-1B18), TIGIT (A15153G), CD160 (BY55) and CD57 (HNK-1) (BioLegend, San Diego, CA; diluted 1:50); CD279/PD-1 (PD1.3.1.3); CD223/LAG-3 (REA351), CD366/TIM-3 (F38-2E2) and CD152/CTLA-4 (BNI3) (Miltenyi Biotec., diluted 1:10). MPM cells were stained for immune ICP ligands using the following antibodies: CD274/PD-L1 (29E.2A3), CD273/PD-L2 (24 F.10C12), CD223/LAG-3 (REA351), CD366/TIM-3 (F38-2E2) (Miltenyi Biotec; diluted 1:10). Cells were analyzed using Guava easyCyte flow cytometer (Millipore) and InCyte software [34].

### Patient-derived xenografts (PDXs)

MPM#1 (epithelioid, BAP1^+^) and MPM#7 (sarcomatoid, BAP1^−^) were subcutaneously (s.c.) injected (1 × 10^7^ cells) in the right flank of 6-week-old female NOD SCID-γ (NSG) mice engrafted with human hematopoietic CD34^+^ cells (Hu-CD34^+^; The Jackson Laboratories, Bar Harbor, MA, USA) or in NSG mice (The Jackson Laboratories). Animals were housed (5/cage) under a 12-hour light/dark cycle, with food and water *ad libitum*. Tumor volumes, measured daily with a caliper, was calculated according to (LxW^2^)/2, where L and W were the length and width of the tumor. Animal weights were monitored throughout the study. When tumors reached a volume of 50 mm^3^, animals (*n* = 4/group) were randomized in the following groups: vehicle (0.1 mL saline solution); cisplatin (5 mg/kg) + pemetrexed (100 mg/kg); lurbinectedin (0.18 mg/kg); atezolizumab (10 mg/kg); ecubectedin (1.2 mg/kg); PM54 (0.9 mg/kg); lurbinectedin (0.18 mg/kg) + atezolizumab (10 mg/kg); ecubectedin (1.2 mg/kg) + atezolizumab (10 mg/kg); and PM54 (0.9 mg/kg) + atezolizumab (10 mg/kg).

Compounds were administered intravenously, vehicle and atezolizumab intraperitoneally. Atezolizumab was administered twice/week for six weeks, the other compounds once/week for three weeks. Lurbinectedin, ecubectedin and PM54 were administered at a dose level previously determined as the Maximum Tolerated Multiple Dose (MTMD), defined as the dose level that did not cause severe body weight loss (decrease greater than 20% over two consecutive days) or noticeable signs of systemic toxicity [35]. Animals were euthanized with zolazepam: xylazine (0.2 mL/kg: 16 mg/kg) on day 49 after randomization. Tumors were removed, digested with 1 mg/mL collagenase and 0.2 mg/mL hyaluronidase for 1 h at 37 °C and filtered (70-µm cell strainer) to obtain a single cell suspension. Infiltrating immune cells were collected by centrifugation on Ficoll-Hypaque density gradient, immunostained and quantified as detailed above. Animal care and experimental procedures were approved by the Italian Ministry of Health (#627/2018-PR, 10/08/2018). The maximal tumors’ volume approved as human endpoints were 1000 mm^3^ *±* 10%. Animals were sacrificed before reaching this volume. Tumor sections, fixed in 4% v/v paraformaldehyde, were stained in situ with Cell Death Detection Kit (TUNEL Assay; Roche, Basel, Switzerlan), followed by nuclei counterstaining with DAPI to measure DNA damage, immunostained for calreticulin (NB600-103, Novus Biologicals Toronto, Canada), as index of ICD, STNG (13674, Cell Signalling), p-IRF3 (BIRBORB1860, BioRbyt, Cambridge, UK) and p-IKKα/β (2697, Cell Signalling) as parameters of cGAS/STING pathway activity, or PD-L1 (Ventana SP142, Roche), followed by a peroxidase-conjugated secondary antibody (Dako dilution 1/1000). Sections were examined with a LeicaDC100 microscope.

### Statistical analysis

In vitro results -derived from three independent experiments- were expressed as mean ± SD and analyzed by a two-way analysis of variance (ANOVA) test. Median tumor volume from in vivo PDX experiments were compared using a two-tailed Mann-Whitney *U* test. All statistical analyses were performed using GraphPad Prism v.9.4.1.

## Results

### Ecubectedin and PM54 inhibit the proliferation of patient-derived MPM cells

**Fig. 1:**
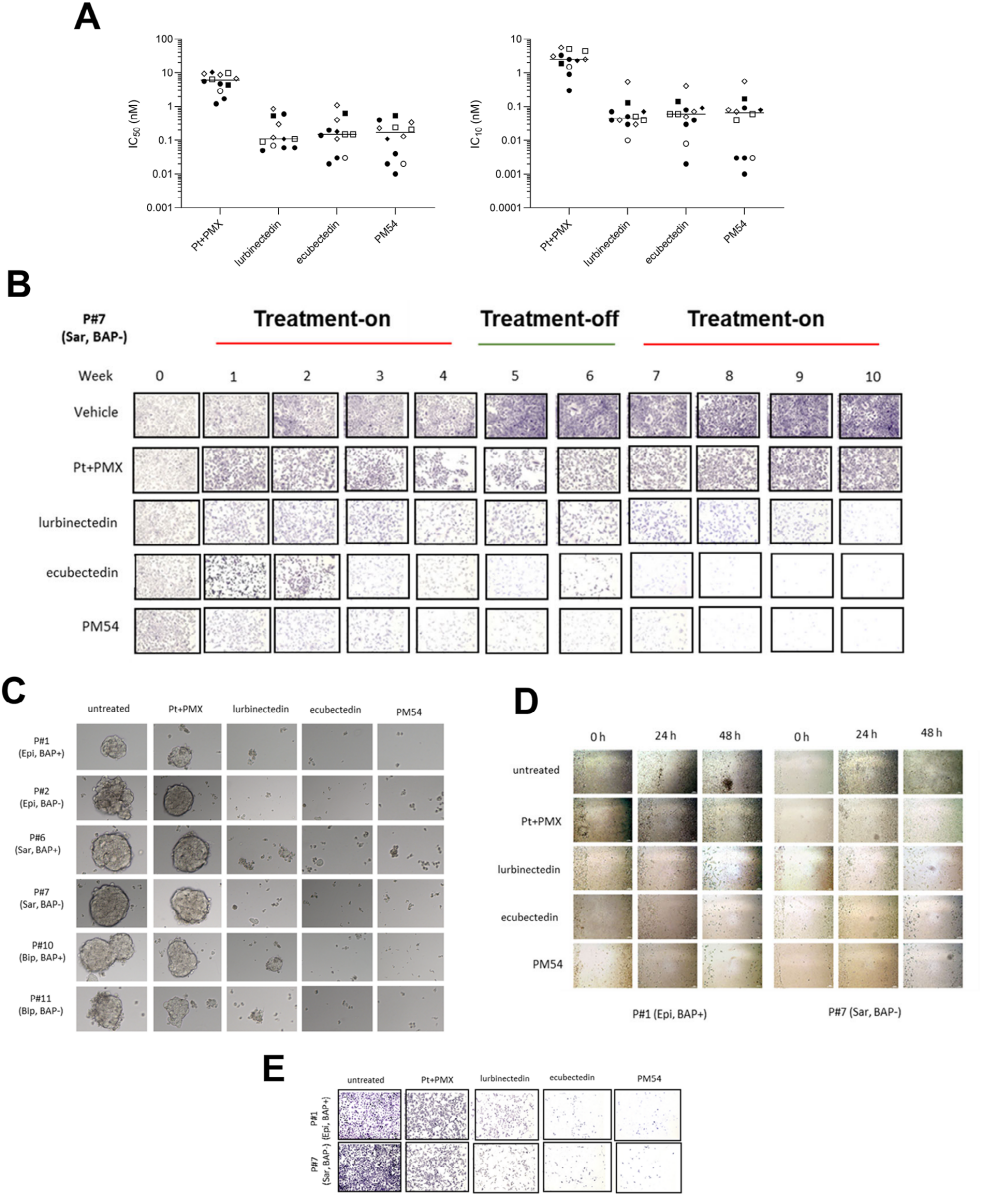
Antiproliferative and anti-invasive effects of lurbinectedin, ecubectedin and PM54 in patient-derived MPM cells. (**A**) In vitro IC_50_ and IC_10_ median values of cisplatin plus pemetrexed (Pt + PMX), lurbinectedin, ecubectedin and PM54 determined in 12 patient-derived histotypes of MPM (epithelioid: square; sarcomatoid: circle; biphasic: diamond) with BAP1 positive (solid symbol) and BAP1 negative (open symbol). Results are mean of 4 independent experiments. (**B**) Representative images of crystal violet staining obtained on long-term (10 weeks) assay with APN#7 cells (sarcomatoid, BAP1-). (**C**). Representative images of the effect induced by the compounds to six different MPM spheroids following 72 h of incubation. Scale bar = 100 μm. (**D**). Representative phase images of migrated cells (scale bar, 100 μm) and (**E**) cells in the lower chamber of APN#1 (epithelioid, BAP1+) and APN#7 (sarcomatoid, BAP1-)

Lurbinectedin, ecubectedin and PM54, as well as Pt + PMX combination induced a concentration-dependent decrease in cell viability in a panel of twelve 2D-cultures of patient-derived MPM, independent of histotype or BAP1 status. The median IC_50_ for Pt + PMX was 6.0 nM and those obtained with lurbinectedin, ecubectedin and PM54 were 0.1 nM, 0.15 nM and 0.17 nM, respectively. Similarly, the median IC_10_ values were 2.5, 0.045, 0.06 and 0.065 nM for Pt + PMX, lurbinectedin, ecubectedin and PM54, respectively (Fig. 1A; Supplementary Table S3; Supplementary Fig. S1-S2). 3 patients (APN 6, 10 and 12) received trabectedin as 2 L treatment, with a poor response to the drug (Supplementary Fig. S1). Accordingly, the patient-derived cell lines did not display higher sensitivity to lurbinectedin or its analogue.

Since most patients treated with chemotherapy relapse even after an initial response acquired resistance [1, 2], we set up long-term experiments, mimicking a 1 L treatment (with drugs administered for 4 weeks at their IC_10_; “drug on” period), followed by a 2-week suspension (“drug off” period) and a second round of 4 weeks with the same treatment. In this setting, secondary resistance usually emerged during the second “drug on” period [36]. Pt + PMX induced heterogeneous effect after the initial treatment period, with cell viability percentages ranging from 32 ± 14% (APN#3; epithelioid, BAP1^+^) to 78 ± 14% (APN#12; biphasic, BAP1^−^). After a 2-week treatment interruption followed by the second treatment period, most cultures experienced a rebound in cell viability, suggesting a strong possibility of induction of resistance to Pt + PMX treatment. Conversely, lurbinectedin and mainly ecubectedin and PM54 induced a continuous and strong decrease in MPM cell viability, unaffected by the 2-week period without treatment, showing median viability values of 12 ± 4%, 3 ± 4% and 5 ± 4%, respectively, regardless of histotype and BAP1 status (Fig. 1B; Supplementary Fig. S3).

Then, we evaluated the tumorigenic in vitro activity on spheroids obtained by six MPM cells representative of different histotypes and BAP1 status. While Pt + PMX combination was ineffective in reducing spheroids growth, lurbinectedin -and, particularly ecubectedin and PM54- induced a strong antitumoral effect, again regardless of histotypes and BAP1 status (Fig. 1C).

### Ecubectedin and PM54 strongly impair invasion, migration and cell cycle of MPM cells

In the wound healing assay (Fig. 1D; Supplementary Figure S4A), Pt + PMX induced a modest effect at 24 (treated vs. untreated migrated cells: 21 ± 4% vs. 45 ± 8%; *p* < 0.05) and 48 (48 ± 9% vs. 73 ± 9%; *p* < 0.05) hours, significantly stronger (*p* < 0.001 vs. untreated cultures) with lurbinectedin (at 24 h: 11 ± 5% vs. 45 ± 8%; at 48 h: 16 ± 8% vs. 73 ± 9%), ecubectedin (at 24 h: 6 ± 4% vs. 45 ± 8%; at 48 h: 9 ± 4% vs. 73 ± 9%) and PM54 (at 24 h: 5 ± 3% vs. 45 ± 8%; at 48 h: 6 ± 3% vs. 73 ± 9%), with the percentages obtained with ecubectedin and PM54 being statistically lower than with lurbinectedin.

Similar result patterns were observed in invasion (Fig. 1E; Supplementary Figure S4B) with highly statistically significant reduction (*p* < 0.001) in the number of migrated cells after incubation with lurbinectedin (108 ± 25 cells/field), ecubectedin (27 ± 6 cells/field) and PM54 (15 ± 5 cells/field), compared to untreated (786 ± 205 cells/field) or Pt + PMX (452 ± 103 cells/field) cells. Notably, ecubectedin and PM54 induced a lower cell migration than lurbinectedin (*p* < 0.001).

After a 24-hour exposure to the compounds at their IC_50_, lurbinectedin, ecubectedin and PM54 increased significantly the percentage of S-phase arrested cells and decreased the percentage of cells in G2/M phase compared to untreated or Pt + PMX-treated cells, suggesting the induction of DNA damage and mitotic arrest. These events are paralleled by increased percentages of sub-G1 apoptotic cells upon lurbinectedin, ecubectedin and PM54 treatment vs. Pt + PMX or untreated cultures (Table 1).

**Table 1:** Cell cycle perturbations of lurbinectedin, ecubectedin and PM54 in a panel of 12 patient-derived MPM cells

Cell cycle phase	Untreated	Pt + PMX	lurbinectedin	ecubectedin	PM54
Sub-G1	4 ± 2	6 ± 3	22 ± 4 ^b^	23 ± 3 ^b^	22 ± 5 ^b^
G0/G1	65 ± 11	69 ± 12	53 ± 12	54 ± 11	51 ± 12
S-phase	4 ± 2	6 ± 3	14 ± 4 ^b^	18 ± 4 ^b^	23 ± 5 ^b^
G2/M	21 ± 5	16 ± 4	9 ± 4 ^a^	8 ± 4 ^a^	5 ± 3 ^a^

Values represent percentage of cells in each phase of the cell cycle

^a^*p* < 0.05; ^b^*p* < 0.001: vs. untreated or Pt + PMX-treated cells

### By causing DNA damage, ecubectedin and PM54 activate STING pathway

**Fig. 2:**
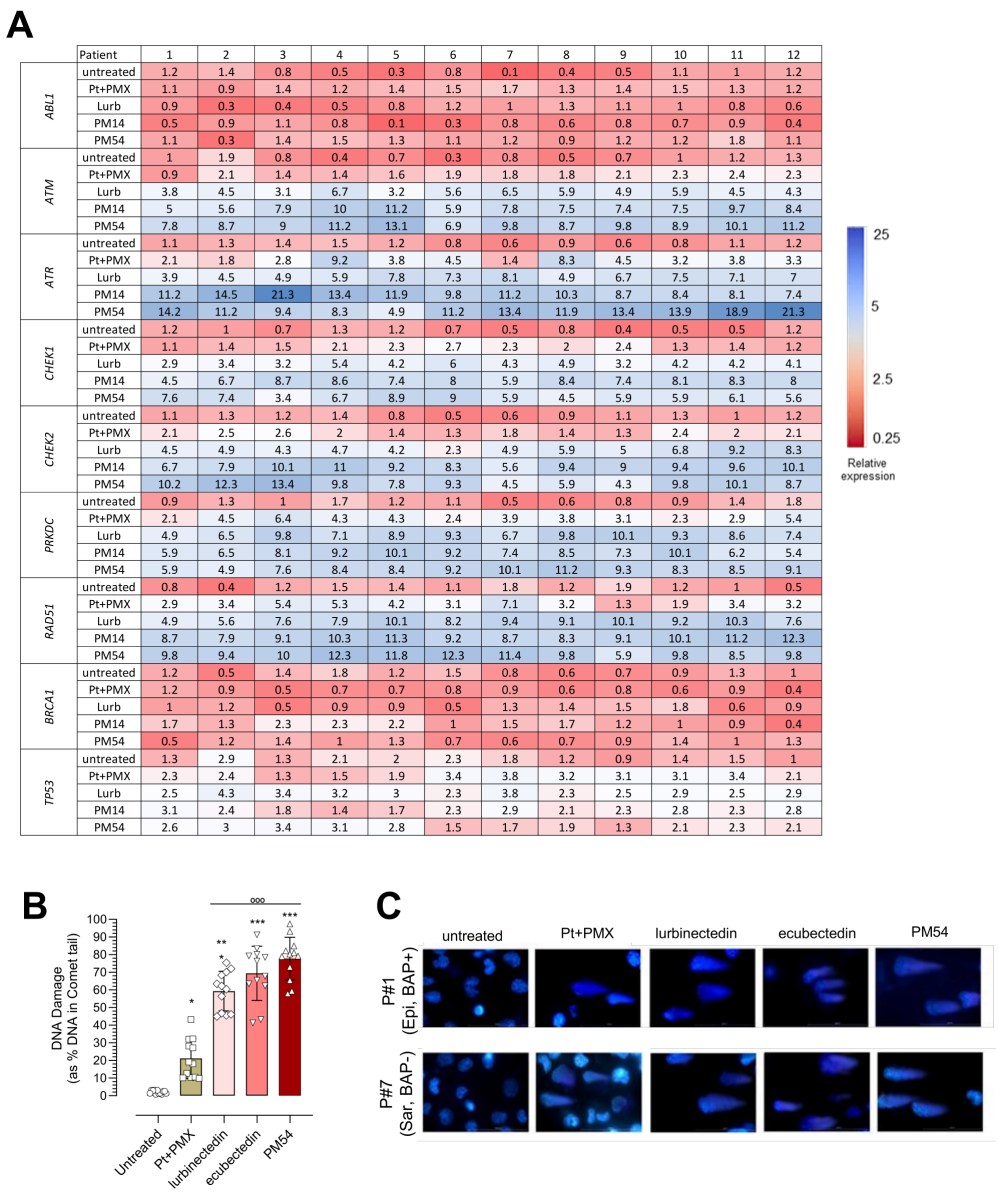
Effects of lurbinectedin, ecubectedin and PM54 on DNA damage and repair machinery in MPM cells. (**A**) The expression pattern of DNA damage genes up-regulated (blue) and down-regulated (red) in MPM cell lines after a 24 h treatment with cisplatin + pemetrexed (Pt + PMX), lurbinectedin (L), ecubectedin and PM54 at their IC_50_ for 24 h, was shown as mRNA abundance versus a pool of housekeeping genes (*n* = 3 independent experiments, in triplicates). (**B**) Histograms showing the percentage of total DNA in the tail of Comet assay in MPM cells treated with Pt + PMX, L, ecubectedin and PM54 at IC_50_ for 24 h. Data are expressed as means ± SD of 12 MPM samples (*n* = 3 independent experiments, in duplicates). *p < 0.05, ***p < 0.001: vs. untreated cells; °°°*p* < 0.001: vs. Pt + PMX. (**C**) Representative Comet assay images of MPM#1 (epithelioid, Bap positive: Epi, BAP+) and MPM#7 (sarcomatoid, BAP1 negative: Sar, BAP-)

The transcriptome profile of MPM cells showed that lurbinectedin, ecubectedin and PM54 down-regulated DNA damage sensors (*ABL1*, *BRCA1* and *TP53*) and up-regulated repair machinery genes (*ATR*, *ATM*, *CHEK1*, *CHEK2*, *PRKDC* and *RAD51*), all unaltered following Pt + PMX treatment (Fig. 2A). Accordingly, lurbinectedin, ecubectedin and PM54 significantly increased DNA damage, regardless of BAP1 status (Fig. 2B-C).

**Fig. 3:**
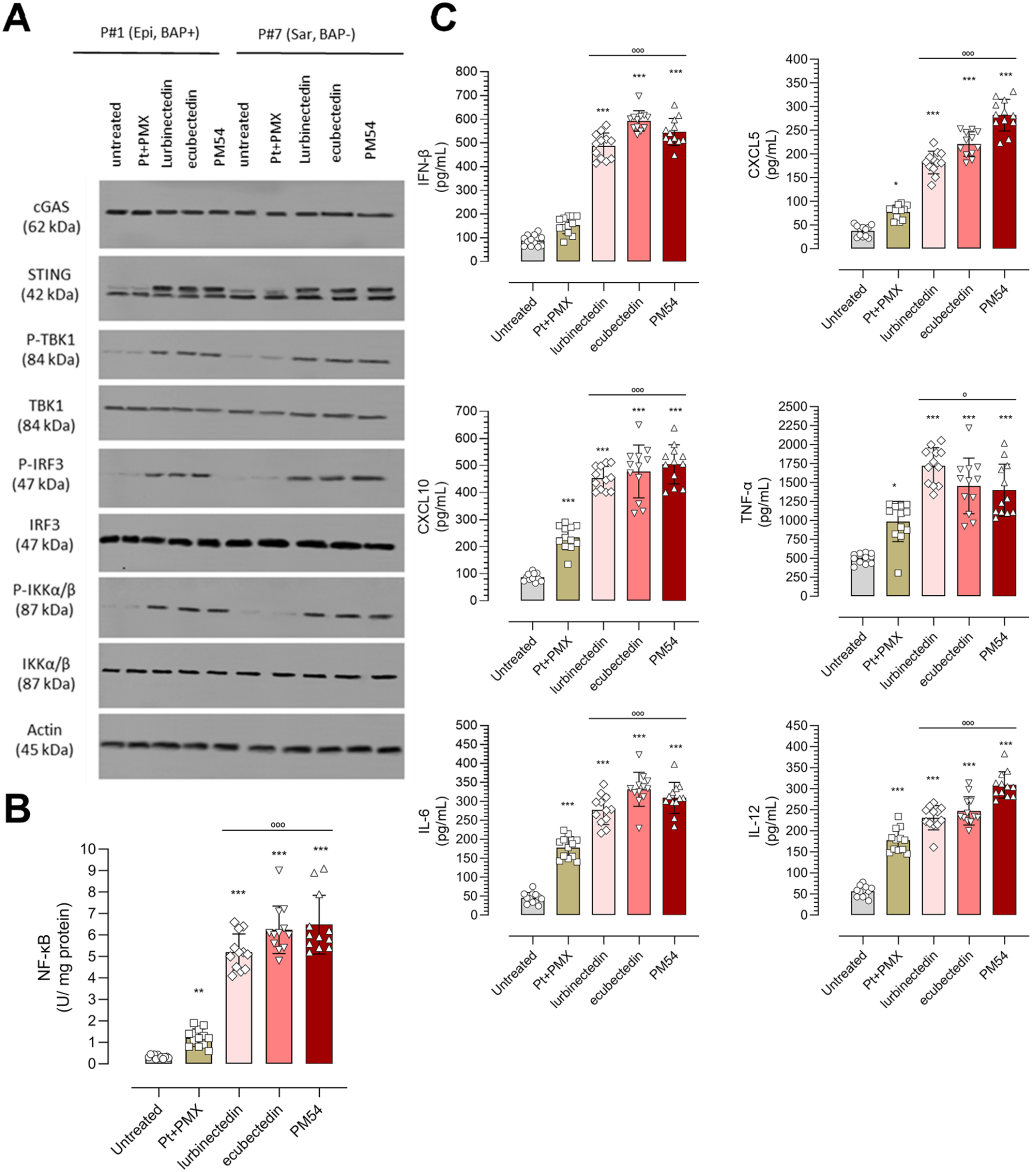
Effects of lurbinectedin, ecubectedin and PM54 on cGAS/STING pathway activation and downstream cytokines in MPM cells. (**A**) Representative immunoblot images of the indicated proteins belonging to the cGAS/STING pathway, in MPM#1 (epithelioid, BAP1 positive: Epi, BAP^+^) and MPM#7 (sarcomatoid, BAP1 negative: Sar, BAP^−^), treated or not (-) with cisplatin + pemetrexed (Pt + PMX), lurbinectedin (L), ecubectedin and PM54 at their IC_50_ for 24 h, actin was used as a loading control. The figure is representative of 1 out of 3 experiments. (**B**) Activation of NF-kB after the treatment indicated in (**A**). Data are expressed as means ± SD of 12 MPM samples (*n* = 3 independent experiments, in duplicates). **p < 0.01, ***p < 0.001: vs. untreated cells; °°°*p* < 0.001: vs. Pt + PMX. (**C**) Pro-inflammatory cytokine/chemokine levels released in the supernatant of MPM cell cultures after the treatments indicated in (**A**). Data are expressed as means ± SD of 12 MPM samples (*n* = 3 independent experiments, in duplicates). *p < 0.05, ***p < 0.001: vs. untreated cells; °°°*p* < 0.001: vs. Pt + PMX

In contrast to Pt + PMX, the three tested agents activated the cGAS/STING pathway, resulting in an increase of STING, phosphorylated TBK1/IRF3 and IKKβ proteins (Fig. 3A; Supplementary Fig. S5), NF-κB activation (Fig. 3B) and proinflammatory cytokines INF-β, TNF-α, CXCL5, CXCL10, IL-6 and Il-12 (Fig. 3C).

### Ecubectedin and PM54 induce immunogenic cell death and immune killing by CD8+T-lymphocytes, reshaping the MPM immune-environment

**Fig. 4:**
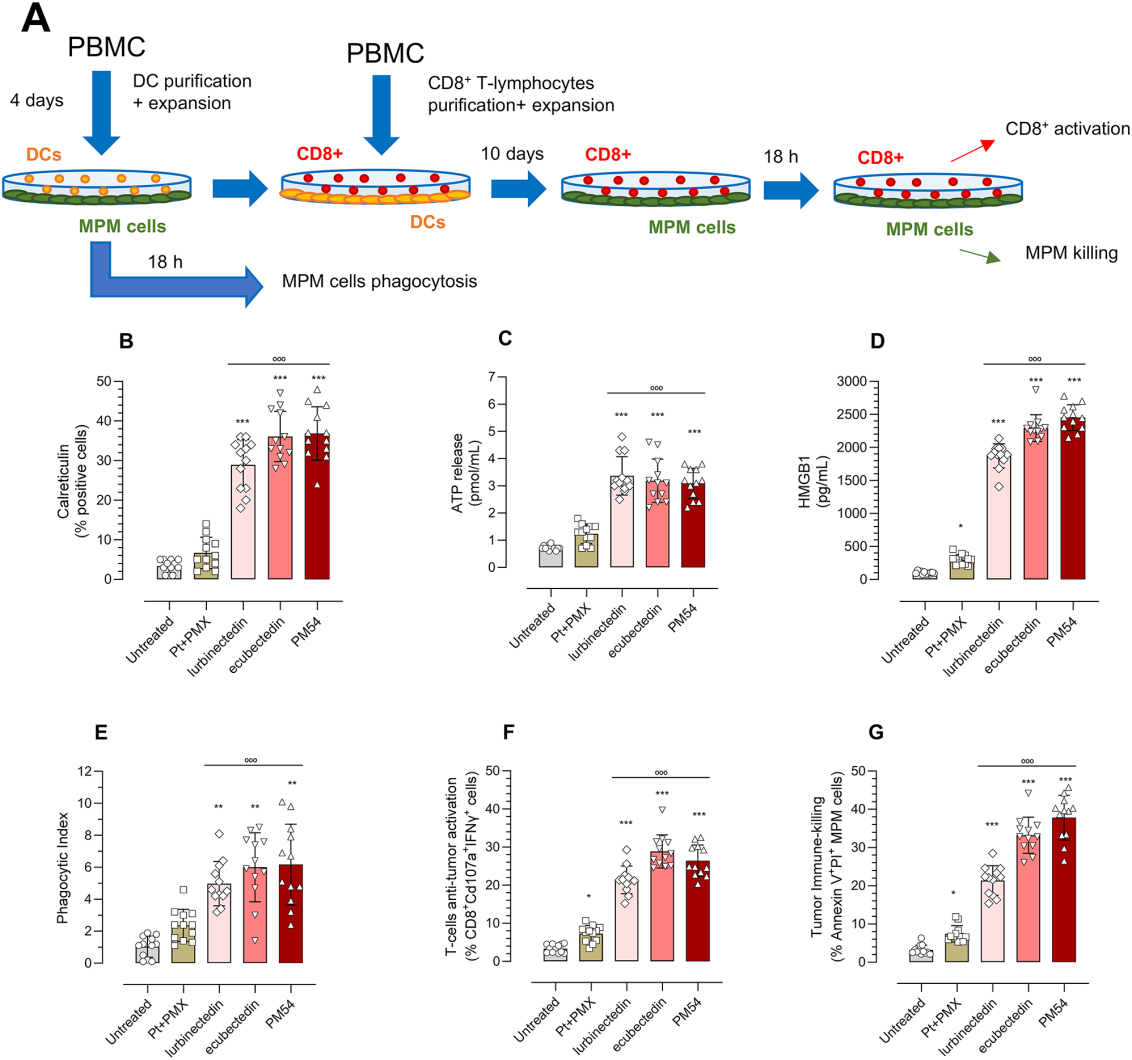
Effects of lurbinectedin, ecubectedin and PM54 on immunogenic cell deaths of MPM cells. (**A**) Workflow of experiment. Dendritic cells (DCs), generated in 4 days from healthy donors PBMC, were co-incubated 18 h with MPM cells, previously grown 24 h in drug-free medium (untreated), cisplatin + pemetrexed (Pt + PMX), lurbinectedin (L), ecubectedin and PM54 at their IC_50_. An aliquot of DC-MPM co-cultures were collected to measure DC-mediated phagocytosis by flow cytometry. An aliquot of DCs that have been in contact with MPM cells was collected and incubated 10 days with CD8^+^T-lymphocytes, obtained from PBMC of the same donor. CD8^+^T-lymphocytes were then collected and co-incubated with the MPM cells of the same patient for 18 h. Finally, CD8^+^T-lymphocytes were collected and analyzed for the activation markers by flow cytometry, MPM cells were stained with Annexin V-FITC/PI to measure necro-apoptotic cells, as index of immune-killing mediated by CD8^+^T-lymphocytes. (**B**) The percentage of cells positive for surface calreticulin (CRT) was measured by flow-cytometry. (**C**) ATP release was measured by a chemiluminescent-based assay. (**D**) HMGB1 release was measured by ELISA. (**E**) Phagocytized MPM cells were counted by flow cytometry. (**F**) Percentage of CD8^+^CD107a^+^INFγ^+^ cells, as index of cytotoxic T-lymphocyte activation. (**G**) Percentage of annexin V-FITC^+^/PI^+^ MPM cells, as index of tumor cells immune killing by CD8^+^T-lymphocytes, measured by flow cytometry. In all panels, data are expressed as means ± SD of 12 MPM samples (*n* = 3 independent experiments, in duplicates). *p < 0.05, ***p < 0.001: vs. untreated cells; °°°*p* < 0.001: vs. Pt + PMX

To investigate whether the three tested agents increased MPM recognition by immune cells, MPM cells co-cultured with DCs for 4 days were set up. DCs were then co-incubated 10 days with CD8^+^T-lymphocytes, tested for their activation and immune-killing potential against MPM cells (Fig. 4A). As shown in Fig. 4B-D), Pt + PMX combination only elicited a small increase in HMGB1, while lurbinectedin, ecubectedin and PM54 increased the three parameters of ICD, i.e. calreticulin exposure, ATP and HMGB1 release. Consistently, lurbinectedin-, ecubectedin- or PM54-treated cells were more phagocytized by DCs (Fig. 4E). Moreover, the CD8^+^T-lymphocytes co-incubated with DCs that have phagocytized these MPM cells demonstrated higher cytotoxic properties, indicated by the high percentage of CD8^+^CD107a^+^INFγ^+^T-cells (Fig. 4F) and immune-killing, indicated by the AnnexinV^+^PI^+^MPM cells (Fig. 4G).

In parallel, we investigated whether the treatment with lurbinectedin, ecubectedin and PM54 changes the immune-suppressive phenotype that is typically induced by MPM cells [28, 34]. To this aim, MPM cells were co-incubated for 5 days with PBMC from healthy donors after a 24-hour treatment period with Pt + PMX, lurbinectedin, ecubectedin and PM54. Pt + PMX did not generate any change in PBMC immune-phenotype, while lurbinectedin and ecubectedin increased NK cells and decreased Treg cells and Mo-MDSC, a decrease that became statistically significant in PM54-treated cells (Supplementary Table S4).

Also, MPM cells treated with lurbinectedin, ecubectedin and PM54 displayed a reduced expression of ICP ligands PD-L1 and LAG-3 (Supplementary Table S5). In CD4^+^T-lymphocytes, ecubectedin and PM54 produced a small reduction of ICP LAG-3 and immunosenescence marker CD57. In CD8^+^T-cells, lurbinectedin, ecubectedin and PM54 reduced PD-1, LAG-3 and CD57, while in NK cells they decreased PD-1 and CD57 (for all parameters: *p* < 0.05), while Pt + PMX did not produce any change (Supplementary Table S5).

### Ecubectedin and PM54 increased the efficacy of atezolizumab in immune-PDX models of MPM

PD-L1 decrease on MPM cells and PD-1 in co-cultured T-lymphocytes constituted the rationale for testing the combination of lurbinectedin, ecubectedin or PM54 with an ICI targeting the PD-1/PD-L1 axis.

**Fig. 5:**
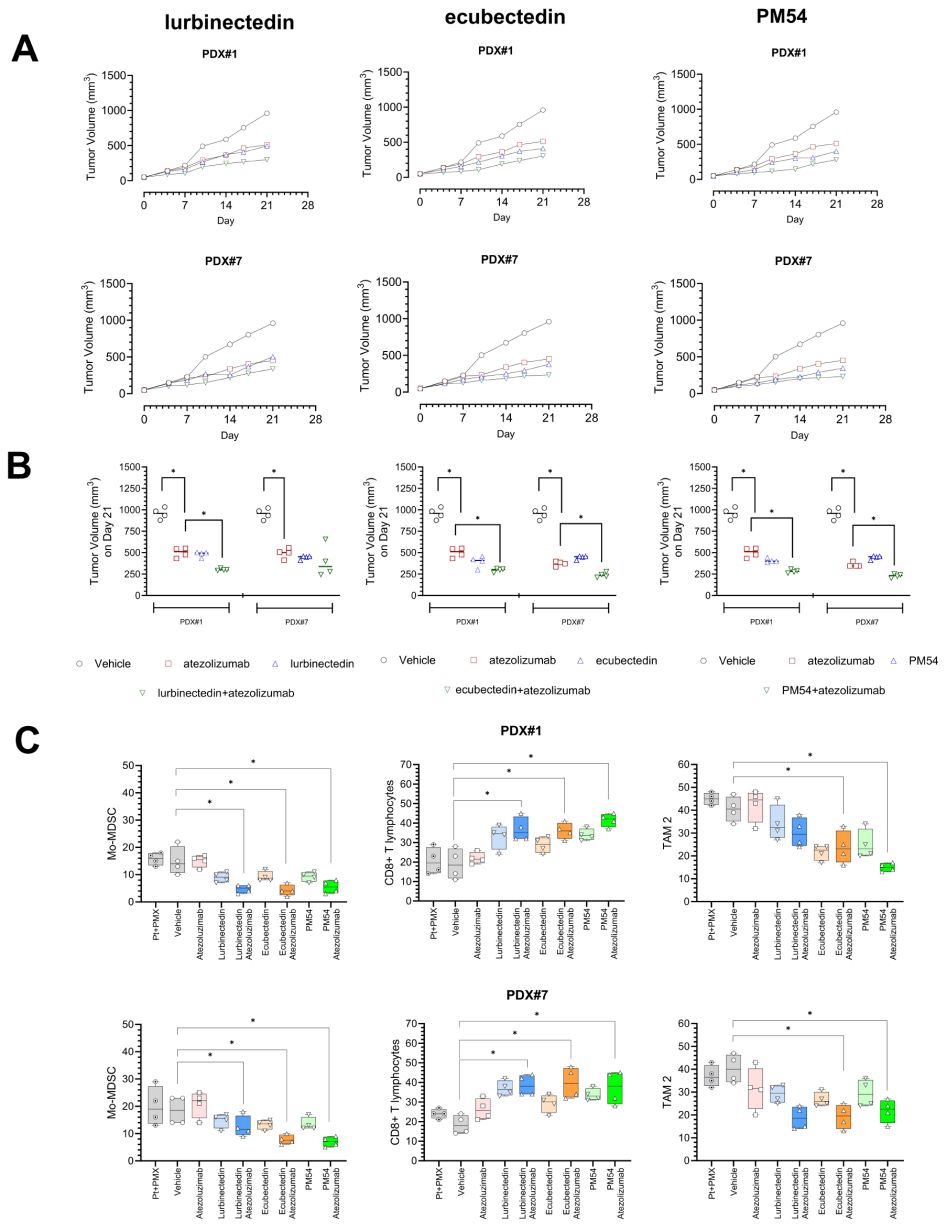
Effects of chemo-immunotherapy treatments based on lurbinectedin, ecubectedin and PM54 in patient-derived immune-xenografts. MPM#1 (epithelioid, BAP1^+^) and MPM#7 (sarcomatoid, BAP1^−^) cells were implanted s.c. in 6-week-old female Hu-CD34^+^ mice and treated as reported under Materials and Methods. (**A**). Tumor volumes growth and (**B**) median tumor volume on day 21 (*n* = 4 mice/group). (**C**) Percentage of Mo-MDSC, CD8^+^T-lymphocytes and TAM2 infiltrating the tumors measured by flow cytometry after tumor excision and dissociation. Pt + PMX, cisplatin plus pemetrexed. For both panels: *p < 0.05

A platform of Hu-NSG mice, bearing an active human immune system, and two MPMs representative of the best patient-derived xenograft (#1 -PDX#-, epithelioid BAP1^+^) and the worst (PDX#7, sarcomatoid, BAP1^−^) case was set up and compared to the same PDX in NSG mice. In both mice strains (NSG and hu-NSG), Pt + PMX was non-effective (versus vehicle) in reducing tumor growth: in NSG mice, 841.5 ± 87.8 mm^3^ versus 926.8 ± 58.9 mm^3^ and 803.8 ± 14.6 mm^3^ versus 950 ± 44.7 mm^3^ for PDX#1 and PDX#7, respectively; in hu-NSG mice, 939.8 ± 56.8 mm^3^ versus 957.8 ± 66.5 mm^3^ and 781.8 ± 14.4 mm^3^ versus 819.3 ± 50.2 mm^3^ for PDX#1 and PDX#7, respectively. In NSG mice, atezolizumab was ineffective (PDX#1: vehicle = 926.8 ± 58.9 mm^3^, atezolizumab = 1030 ± 80.0 mm^3^; PDX#7: vehicle = 950 ± 44.7 mm^3^, atezolizumab = 1098 ± 46.8 mm^3^), as expected, while lurbinectedin, ecubectedin or PM54 resulted in a statistically significant reduction of the final median tumor volume compared to vehicle-treated animals (PDX#1: vehicle = 926.8 ± 58.9 mm^3^, lurbinectedin = 542.5 ± 16.6 mm^3^, ecubectedin = 513.5 ± 24.1 mm^3^ and, PM54 = 520 ± 11.7 mm^3^; PDX#7: vehicle = 950 ± 44.7 mm^3^, lurbinectedin = 584.8 ± 16.8 mm^3^, ecubectedin = 462.8 ± 36.0 mm^3^ and, PM54 = 418 ± 25.7 mm^3^, *p* < 0.05). In hu-NSG mice xenografts, lurbinectedin, ecubectedin and PM54 also induced a strong antitumor effect, similar to that observed in NSG mice, but atezoluzimab treatment induced a statistically significant reduction in tumor volume compared to vehicle-treated mice, an effect significantly enhanced by combining atezoluzimab with lurbinectedin, ecubectedin and PM54 (Fig. 5A). Accordingly, an appreciable DNA damage, evaluated by TUNEL staining, was detected in PDX#1 and PDX#7 implanted in hu-NSG mice treated with lurbinectedin, ecubectedin and PM54, alone and in combination with atezolizumab, but not in case of treatment with Pt + PMZ or atezolizumab alone (Supplementary Fig. S6A). Calreticulin, the key marker of ICD, was not changed (Supplementary Fig. S6B), likely because immunohistochemistry did not discriminate the preponderant, total amount of calreticulin and the plasma membrane associated-calreticulin, which represents a minor amount but induces ICD. By contrast, lurbinectedin, ecubectedin and PM54 strongly activated intratumor cGAS/STING pathway (Supplementary Fig. S6C) and decreased PD-L1 (Supplementary Fig. S6D) compared to untreated or Pt + PMX treated mice. Furthermore, we detected significant increase in the anti-tumor CD8^+^T-lymphocytes and NK cells, and a decrease in the immune-suppressive populations Mo-MDSC and TAM2 (Fig. 5B) infiltrating the tumor in xenografts treated with atezolizumab combined with lurbinectedin, ecubectedin and PM54, while Pt + PMX did not change significantly compared to animals treated with vehicle. None of the treatments induced changes in CD4^+^T-lymphocytes, Treg cells, TAM1 or Gr-MDSC in PDX#1, but they elicited such changes in the most clinically aggressive PDX#7 (Supplementary Fig. S7; Supplementary Table S7). Although lurbinectedin, ecubectedin and PM54 produced similar effects when used as single agents, the widest changes in immune-infiltrating cells were observed when combined with atezolizumab.

## Discussion

For approximately two decades, Pt + PMX has been a well-established standard 1 L treatment for advanced stage MPM [37]. The advent of immunotherapy paved the way to a series of 1 L and 2 L clinical studies based on ICIs. The limited benefit of immunotherapy, mainly in sarcomatoid histotype, and the lack of effective 2 L and 3 L treatment for MPM, prompted us to investigate the potential of lurbinectedin, effective against patient-derived MPM cells [38], and two derivatives, as single agents or in combination with ICIs, in ex vivo and PDX models of MPM.

With an IC_50_ in the low nanomolar range, lurbinectedin, ecubectedin and PM54 were far more cytotoxic than Pt + PMX in MPM cells and disabled the formation of tumor spheres independently from histological subtype or status of BAP1, whose deletion is a critical step in the malignant transformation of mesothelial cells [10]. Also, all compounds strongly reduced MPM cell migration and invasion. Such decrease may be due to reduced cell proliferation and increased apoptosis elicited by ecteinascidin synthetic analogues. Indeed, many DNA-damaging agents that induce cell cycle arrest and apoptosis, subsequently decrease cell viability and migration. For instance, the 1-deoxynojirimycin derivative C6, which produces a significant S-phase arrest of lung, colon and cervical cancer cell lines, also decreases cell migration [39]. Similarly, capsaicin, which increases the percentage of MPM cells arrested in S-phase, also reduces cell migration and invasion in wound-healing assays and Transwell migration assay [40], i.e. the same assays used in the present work. The increased apoptosis and the reduced migration have been attributed to the inhibition of AKT and ERK1/2 signaling in MPM cells [40]. In small cell lung cancer cell lurbinectedin is known to inhibit AKT signaling [41]. Based on this evidence, we hypothesize that ecteinascidin synthetic analogues exert multiple mechanisms on MPM cells, inducing DNA damage and cell cycle arrest, increasing apoptosis, likely via AKT inhibition, and finally leading to decreased cell viability and migration.

This is of paramount importance because MPM’s attitude to spread into the pleural cavity makes surgical resection impossible and determines a high percentage of recurrence even after multimodal therapy [42]. No pharmacological agents block MPM cell invasion: lurbinectedin, PM54 and ecubectedin may represent the first prototypical drugs in this perspective, once tested in proper orthotopic MPM animal models.

Another relevant aspect of these drugs is their ability to induce long-term control of cell proliferation. Most MPM patients become resistant to 1 L treatment [37] and no 2 L treatments have been successful at clinical level so far [6]. Long-term proliferation assays based on a 4-week period of drug exposure followed by a 2-week interruption and a second 4-week exposure to the previous drugs, showed a great variability in the efficacy of Pt + PMX among patient-derived cell lines, with only 2 cultures out of 12 showing a growth reduction by the second exposure. Remarkably, lurbinectedin, ecubectedin and particularly PM54 showed no re-growth during the treatment break period and the second exposure in all samples. We cannot exclude that the effect of ecteinascidins was caused by an increased cell death – stronger than the effect of Pt + PMX – that can even produce a certain number of persister cells. However, the striking decrease in viable cells in the long term indicates that persisting cells are irreversibly damaged and unable to proliferate and/or that the cells do not acquire resistance towards these agents.

The main mechanism of cytotoxicity induced by lurbinectedin, ecubectedin and PM54 is the DNA damage, an event that has two distinct but interconnected effects: the direct killing of MPM cell and the increased priming of MPM cell for immune killing. Besides arresting cell proliferation, drugs produced a large amount of double-strand fragmented DNA, not prevented by the increase in specific genes of DNA repair system. In MPM, ATR/CHEK1, ATM/CHEK1 and ATM/CHEK2, activation has been diversely related to increased or decreased sensitivity to Pt [43]. In analyzed patient-derived cell lines in this study, the scarcely effective combination Pt + PMX produced minor variations in these genes, while lurbinectedin, ecubectedin or PM54 increased them, notwithstanding a certain inter-patient variability. Despite such increase, MPM cells were unable generating an effective DNA repair, because lurbinectedin, ecubectedin and PM54 decreased at the same time the DNA-damaged sensors *ABL1* and *BRCA1*. The high number of double-stranded DNA fragments present within the MPM dying cells, activated the cGAS/STING pathway. To the best of our knowledge, this is the first study that reports an activation of cGAS/STING pathway in MPM, exerted by a clinically approved pharmacological agent. Previous findings indicated that STING and its transducer IRF3 were highly expressed in human MPM samples, particularly in non-epithelioid phenotype [44]. MPM is strongly responsive to synthetic activators of cGAS/STING pathway that increase the production of CXCR3 in tumor cells and cancer-associated fibroblasts, resulting in the recruitment and activation of NK cells and mesothelin-targeted chimeric antigen receptor (CAR)-NK cells [45].

In colon cancer the activation of cGAS/STING pathway upon the treatment with the DNA-damaging agent oxaliplatin induced ICD of tumor cells, followed by cancer cell phagocytosis by DCs and cytotoxic CD8^+^T-cells anti-tumor response [46]. ICD elicited by DNA damaging agents is a common event in tumors with defects in mismatch repair and/or high genetic instability, such as colon cancer [47], where the tumor mutation burden and immunogenicity are easily increased. Conversely, this process is unlike in tumors characterized by high DNA stability and low immunogenicity, such as MPM. Transforming an immunogenically “cold tumor” into a “hot tumor” is still an open challenge in the era of immunotherapy [48]. Thanks to their ability of producing irreversible double-stranded DNA fragments that activates the cGAS/STING pathway, lurbinectedin as well as ecubectedin and PM54 emerged as powerful inducers of ICD in the “cold” MPM, in line with previous findings demonstrating that lurbinectedin is an ICD inducer [49]. In line with what was described for cGAS/STING pathway agonists [45], lurbinectedin, ecubectedin and particularly PM54 increased in CD8^+^T-cells and NK cells in MPM-PBMC co-cultures. Of note, we also observed a decrease in immune-suppressive populations such as Treg cells and Mo-MDSC. Both Treg and Mo-MDSC are abundant in MPM microenvironment [34, 50] and associated with poor prognosis [51], because they limit the proliferation and cytotoxic activity of tumor infiltrating lymphocytes (TILs) [34, 52].

Intriguingly, MPM cells with high levels of IRF3 have been related to high levels of ICP ligands PD-L1, LAG-3, TIM-3 and CTLA-4 on MPM cells [44], providing a putative explanation for the immune-anergic profile of TILs and failure of ICI-based treatment. On the one hand, lurbinectedin, ecubectedin and PM54 activated cGAS/STING pathway and on the other hand, they decreased the levels of PD-L1 and LAG-3 on MPM cells. We suggest that the damage elicited by these agents impairs multiple cellular functions, including gene transcription, protein translation and surface receptor recycling: the decrease in ICP ligands on MPM cell surface may be the consequence of these events. In addition, the decrease in Treg further relieves the immune-anergy attitude of T-lymphocytes [52]. In line with this observation, we found decreased amounts of PD-1 and LAG-3 on cytotoxic populations (CD8^+^T-lymphocytes and NK cells), associated with a decrease in CD57, a typical immune senescence marker that predicts low response to ICIs in lung cancer [53]. The effects observed in ex-vivo systems were phenocopied in PDXs, where ecteinascidin synthetic analogues increased DNA damage, cGAS/STING pathway and CD8^+^T-lymphocytes, i.e. the immune cells recruited upon ICD induction, and decreased PD-L1 intratumor levels.

The decrease in ICP/ICP ligands and CD57 levels, the increased ratio between anti-tumor/immune-suppressive populations set the rationale bases for the combination of lurbinectedin or ecubectedin and PM54 with ICI. We chose the combination with PD-L1 inhibitor atezolizumab, considering this combination a potentially promising 1 L treatment in MPM.

This combination did not offer additional benefit as compared to lurbinectedin, ecubectedin or PM54 administered alone in immune-deficient mice, but it showed a significantly stronger effect than atezolizumab alone in immunocompetent mice. The phase 3 BEAT-meso study did not show a survival benefit obtained by atezolizumab as 1 L in MPM patients [54], but our study demonstrated for the first time that the combination of atezolizumab and ecteinascidin improved atezolizumab’s efficacy in both epithelioid and sarcomatous MPM-PDX, offering a new approach to include the ICI atezolizumab in the arena of the anti-MPM treatments that goes beyond the current and unsatisfactory immunotherapy used in MPM, based on nivolumab and ipilimumab. Atezolizumab is currently regarding as a promising alternative, as demonstrated by the recently started Immuno-MESODEC phase I/II trial evaluating the feasibility and safety of chemo-immuno-therapy based on Pt + PMX + Atezolizumab, associated with vaccination based on autologous Wilms’ tumor 1 mRNA-electroporated DCs [55].

The present study also indicates that the anti-tumor effects of the combination of ecteinascidins and atezolizumab are in part due to the engagement of the host immune system against the tumor.

Indeed, the analysis of the immune-infiltrate recapitulated the immune-phenotype of MPM-PBMC co-cultures, confirming that lurbinectedin, ecubectedin and PM54 increased CD8^+^T-cells and NK cells, and decreased Mo-MDSCs. Additionally, they reduced M2-polarized macrophages, further eliminating a typical pro-tumoral population of MPM microenvironment [34, 50], necessary for MPM tumorigenesis and progression [56]. Although a limitation of our study is the small size of patient-derived cell lines and PDXs, our collection of primary MPM cells is representative of the different histologies and BAP1 mutational status, and the PDXs platform included the best- and worst-case MPM scenario.

## Conclusion

In summary, the study revealed that lurbinectedin, ecubectedin, and PM54 induce significant DNA damage leading to direct MPM cell death, enhance immune recognition by innate immunity cells, and alter the immune environment of MPM by favoring anti-tumor over pro-tumor populations. These findings suggest the potential for clinical evaluation of these agents, either individually or in combination with an ICI, in MPM patients.

## Electronic Supplementary Material

Below is the link to the electronic supplementary material.

Supplementary Material 1: Figure S1. Dose-response viability curve of cells of single MPM samples. Figure S2. Representative crystal violet staining of single MPM samples. Figure S3. Long-term effects of lurbinectedin, ecubectedin and PM54 on MPM cells proliferation. Figure S4. Quantification of the effects of lurbinectedin, ecubectedin and PM54 on cell invasiveness and migration. Figure S5. Densitometric analysis of the immunoblotting of Fig. 3B. Figure S6. Immunohistochemical analysis of the MPM1 and MPM7 implanted in Hu-NSG mice. Figure S7. Tumor-infiltrating immune-populations infiltrating MPM unaffected by lurbinectedin, ecubectedin and PM54. Table S1. MPM primary samples, clinical features and treatments. Table S2. MPM primary samples histological characterization. Table S3. IC50 (nM) of cisplatin plus pemetrexed, lurbinectedin, ecubectedin and PM54 in a panel of 12 patient-derived MPM cells. Table S4. Immunophenotype of PBMC after 5 day-incubation with MPM cells. Table S5. ICP/ICP ligands and immune-senescence expression on T-lymphocytes and MPM cells, after lurbinectedin, ecubectedin and PM54. Table S6. Quantification of the immune-infiltrating cells in excised MPM1 and MPM7 implanted in Hu-NSG mice.

## Data Availability

All data generated or analyzed during this study are included in this published article and its supplementary data files.

## Abbreviations

MPM: Malignant pleural mesothelioma
OS: Overall survival
Pt + PMX: Platinum + pemetrexed
ICI: Immune checkpoint inhibitor
1L: First line
2L: Second line
3L: Third line
BAP1: BRCA1-associated protein
TAM: Tumor associated macrophage
SCLC: Small cell lung cancer
NK: Natural killer
Treg: T-regulatory
APN: Anonymous patient number
FBS: Fetal bovine serum
PS: Penicillin-streptomycin
PI: Propidium iodide
ICD: Immunogenic cell death
CRT: Calreticulin
HMGB1: High-mobility group box-1
DC: Dendritic cells
PBMC: Peripheral blood mononuclear cells
GR-MDSC: Granulocyte-derived myeloid derived suppressor cells
Mo-MDSC: Monocyte-derived myeloid derived suppressor cells
ICP: Immune checkpoint
NSG: NOD SCID-γ
PDX: Patient-derived xenograft
CAR: Chimeric antigen receptor
TILs: Tumor infiltrating lymphocytes
