# Supplementary material for: Ecteinascidin synthetic analogues: a new class of selective inhibitors of transcription, exerting immunogenic cell death in refractory malignant pleural mesothelioma
Source: J Exp Clin Cancer Res. 2024 Dec 21;43:327. doi: 10.1186/s13046-024-03253-y (PMC11662834; doi:10.1186/s13046-024-03253-y)
Supplement: Supplementary file 1 — Supplementary Material 1: Figure S1. Dose-response viability curve of cells of single MPM samples. Figure S2. Representative crystal violet staining of single MPM samples. Figure S3. Long-term effects of lurbinectedin, ecubectedin and PM54 on MPM cells proliferation. Figure S4. Quantification of the effects of lurbinectedin, ecubectedin and PM54 on cell invasiveness and migration. Figure S5. Densitometric analysis of the immunoblotting of Fig. 3B. Figure S6. Immunohistochemical analysis of the MPM1 and MPM7 implanted in Hu-NSG mice. Figure S7. Tumor-infiltrating immune-populations infiltrating MPM unaffected by lurbinectedin, ecubectedin and PM54. Table S1. MPM primary samples, clinical features and treatments. Table S2. MPM primary samples histological characterization. Table S3. IC50 (nM) of cisplatin plus pemetrexed, lurbinectedin, ecubectedin and PM54 in a panel of 12 patient-derived MPM cells. Table S4. Immunophenotype of PBMC after 5 day-incubation with MPM cells. Table S5. ICP/ICP ligands and immune-senescence expression on T-lymphocytes and MPM cells, after lurbinectedin, ecubectedin and PM54. Table S6. Quantification of the immune-infiltrating cells in excised MPM1 and MPM7 implanted in Hu-NSG mice. [file 13046_2024_3253_MOESM1_ESM.docx]

# SUPPLEMENTARY MATERIALS

**Supplementary Figures**

**
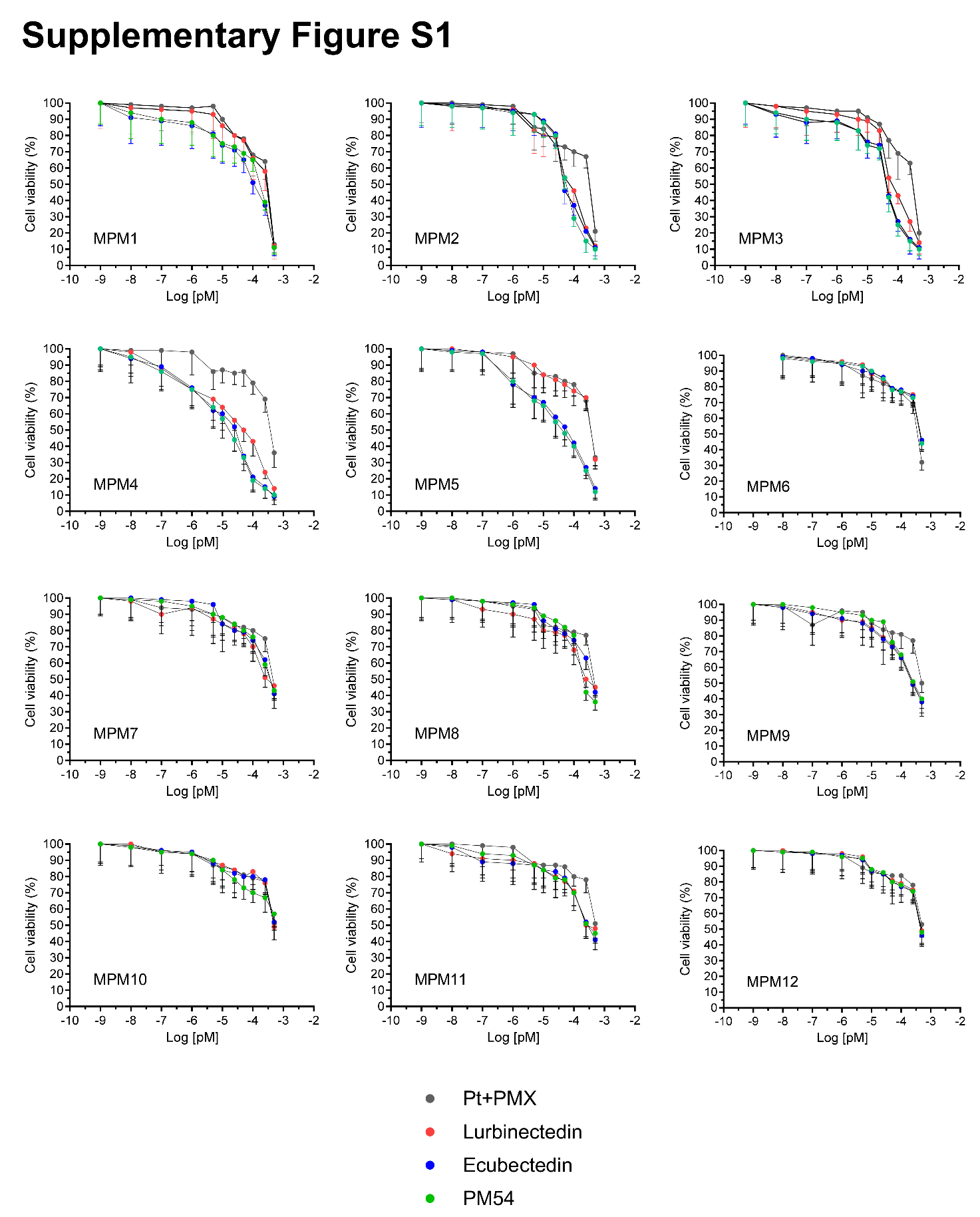
**

**Supplementary Figure S1. Dose-response viability curve of cells of single MPM samples.** Dose-response viability curve of cells of 12 MPM samples after 72h treatments with fresh medium (0) or increasing concentrations. 1 nM–100 µM of cisplatin+pemetrexed (Pt+PMX), lurbinectedin (L), ecubectedin and PM54 (n = 4).

**
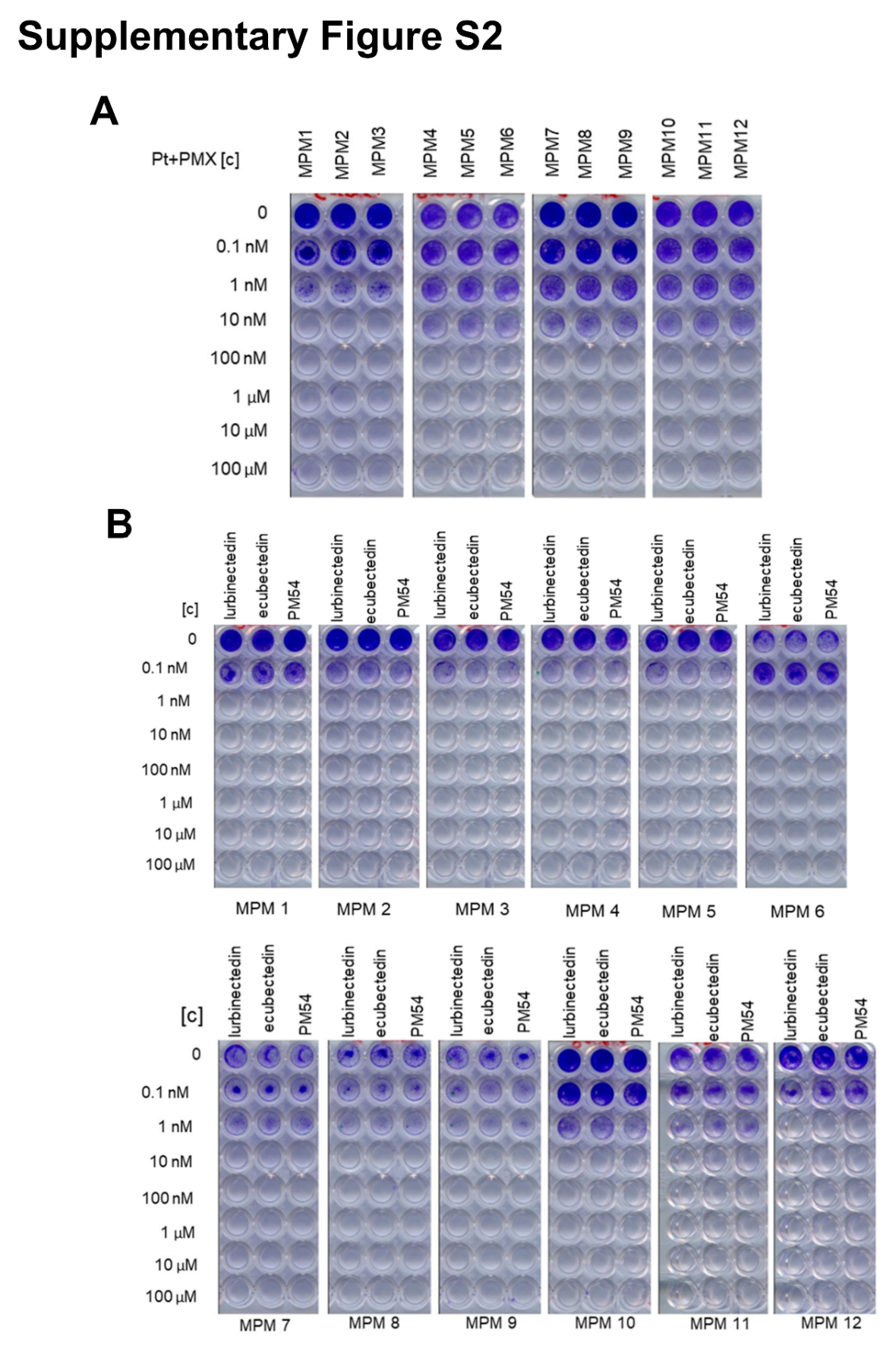
**

**Supplementary Figure S2. Representative crystal violet staining of single MPM samples.** Representative images of viable (crystal violet stained) cells of 12 MPM samples after 72h treatments with fresh medium (0) or increasing concentrations. 1 nM–100 µM of cisplatin+pemetrexed (Pt+PMX) (**A**), lurbinectedin (L), ecubectedin and PM54 (**B**). The images are representative of 1 out of 4 experiments.

**
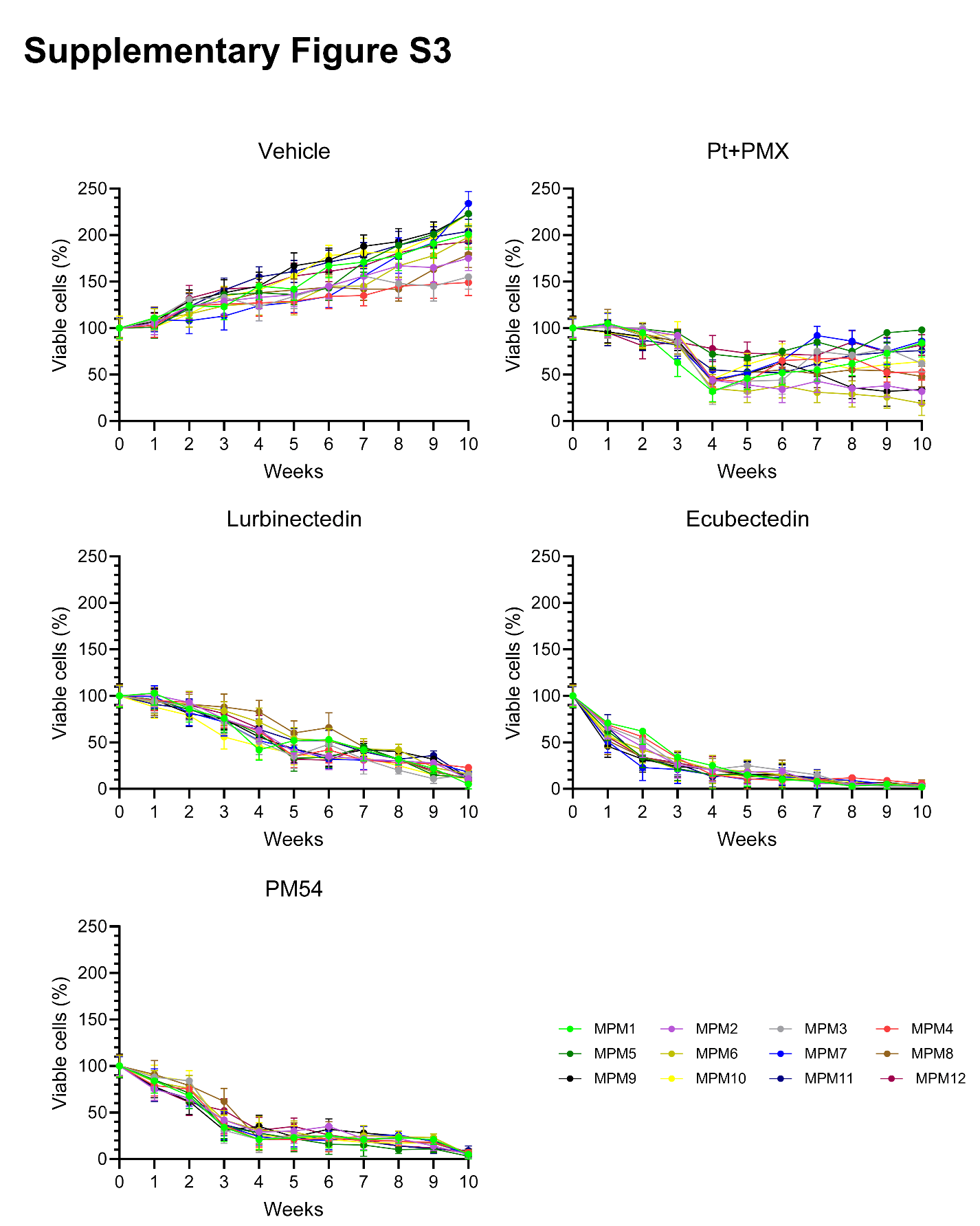
**

**Supplementary Figure S3.** **Long-term effects of lurbinectedin, ecubectedin and PM54 on MPM cells proliferation.** Dose-response curves of MPM 2D-cultures treated for 4 weeks, with cisplatin+pemetrexed (Pt+PMX), lurbinectedin, ecubectedin and PM54, administered once/week at IC_10_, then grown in drug-free medium (drug holiday) for 2 weeks, treated with a second 4-week cycle. Data are means+SD (n= 3 independent experiments, in quadruplicates).

**
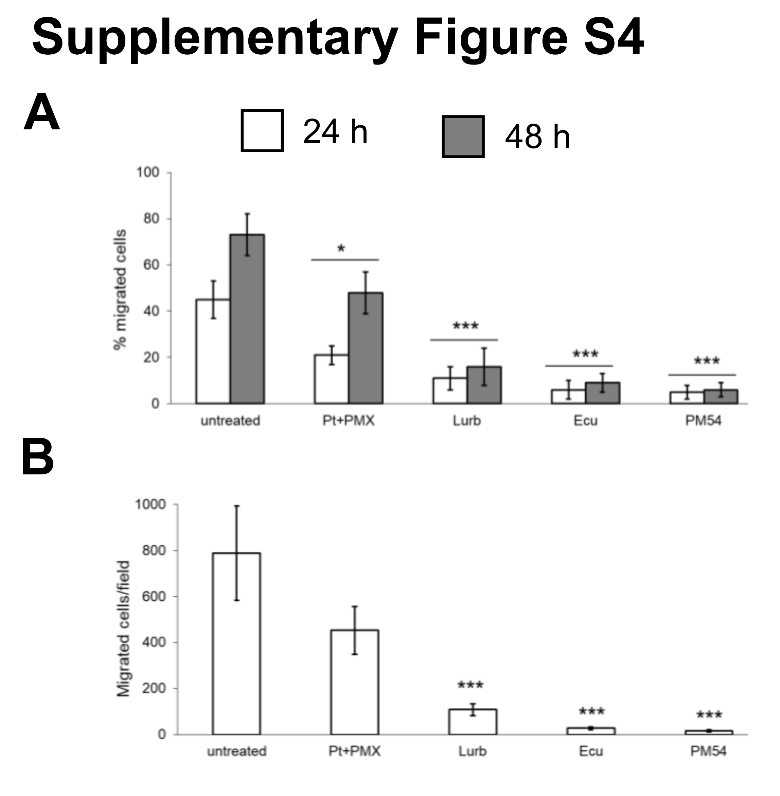
**

**Supplementary Figure S4.** **Quantification of the effects of lurbinectedin, ecubectedin and PM54 on cell invasiveness and migration.** (A) Quantification of cell migration (refer to Figure 1D) calculated using ImageJ software, and (B) quantification of cell invasion (refer to Figure 1E), performed by spectrophotometric quantification of cells migrated in the lower insert of Transwell device and stained by crystal violet. Data are expressed as means±SD (n=3 independent experiments, in duplicates). Pt-PMX: cisplatin+pmetrexed; Lurb: lurbinectedin; Ecu: ecubectedin. *p<0.05, ***p<0.001: vs untreated cells.

**
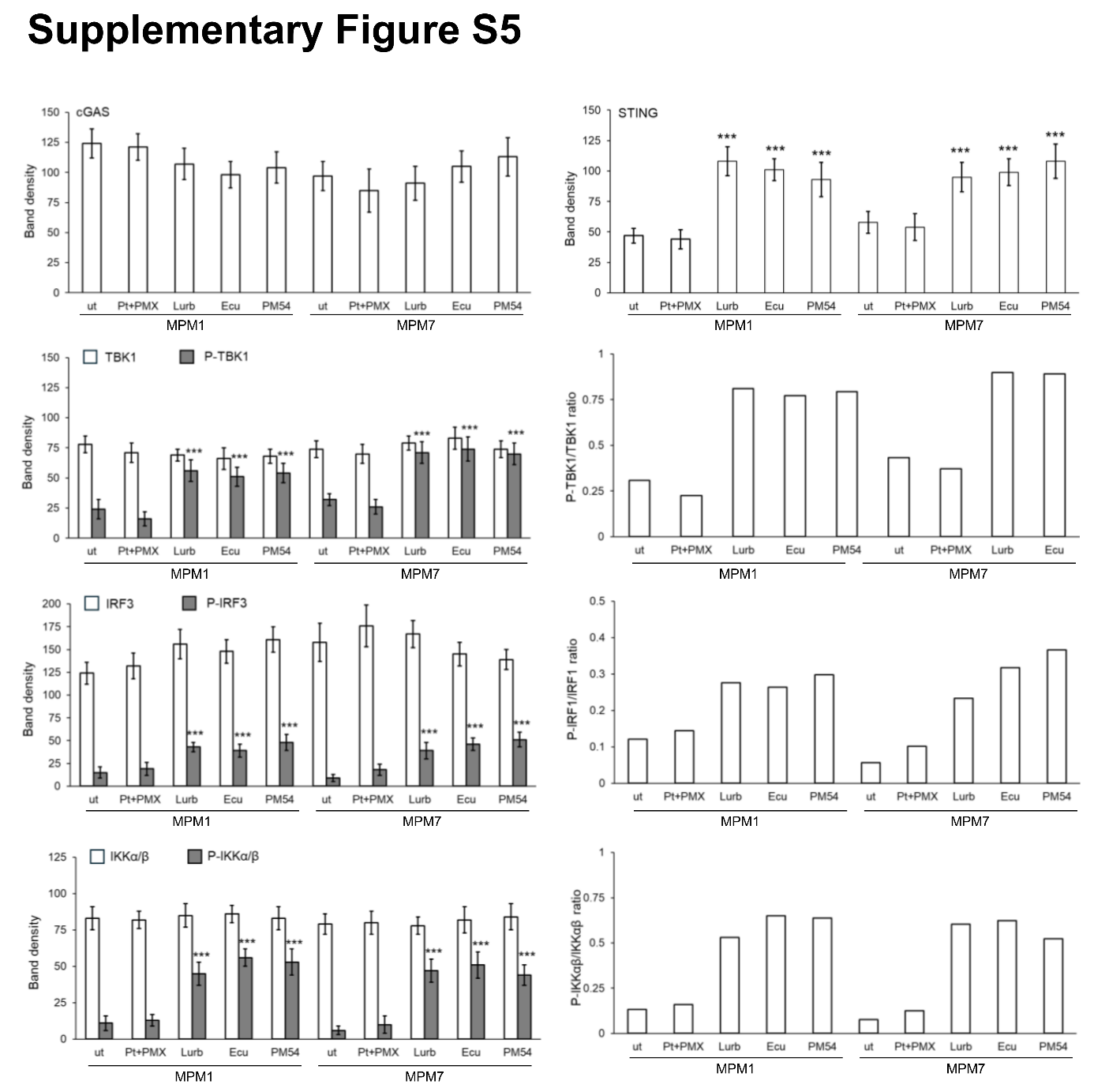
**

**Supplementary Figure S5. Densitometric analysis of the immunoblotting of Figure 3B.** Quantification of the immunoblot of Figure 3B, calculated using ImageJ software. Data are expressed as means±SD (n=3 independent experiments). p-TBK1/TBK1 ratio, p-IRF1/IRF1 ratio, p-IKKαβ/ IKKαβ ratio are the mean ratio between phosphorylated and total proteins. Ut: untreated cells; Pt-PMX: cisplatin+pmetrexed; Lurb: lurbinectedin; Ecu: ecubectedin. ***p<0.001: vs untreated cells.

**
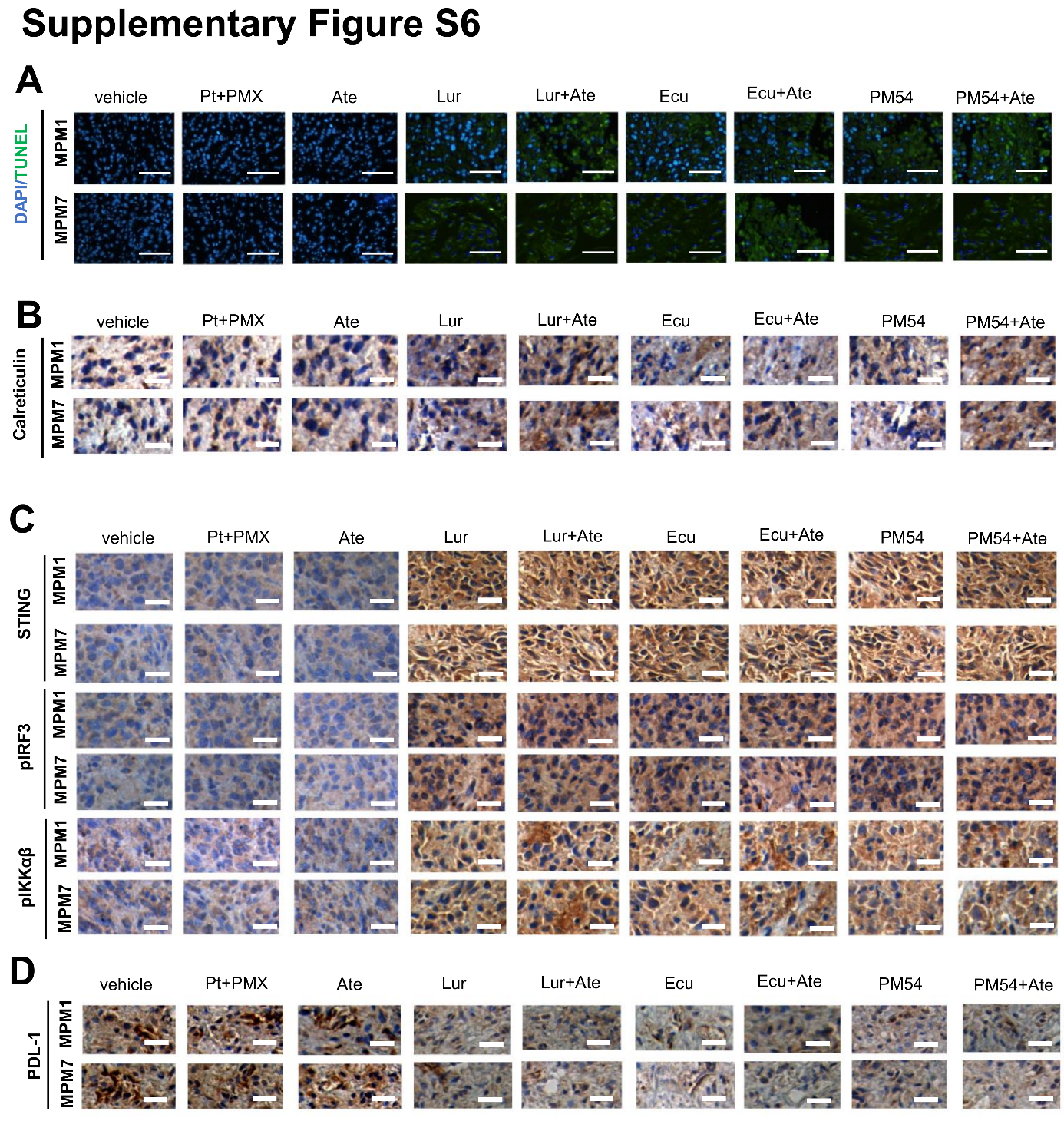
**

**Supplementary Figure S6. Immunohistochemical analysis of the** **MPM1 and MPM7 implanted in Hu-NSG mice.** Representative staining of (A) TUNEL as index of DNA damage, (B) calreticulin as index of immunogenic cell death, (C) STING, phospho-IRF3 and phosphor-IKKα/β as indexes of cGAS/STING pathway, (D) PD-L1 in MPM#1 (epithelioid, BAP1+) and MPM#7 (sarcomatoid, BAP1-) tumors implanted in 6-week-old female Hu-CD34+ mice and treated as reported under Materials and Methods. Objective: 20× (panel A), 20× (panels B-D); ocular: 10×. Bar=100 µm (panel A), 100 µm (panels B-D). Pt+PMX: pemetrexed; Ate: atezolizumab; Lur: lurbinectedin; Ecu: ecubectedin


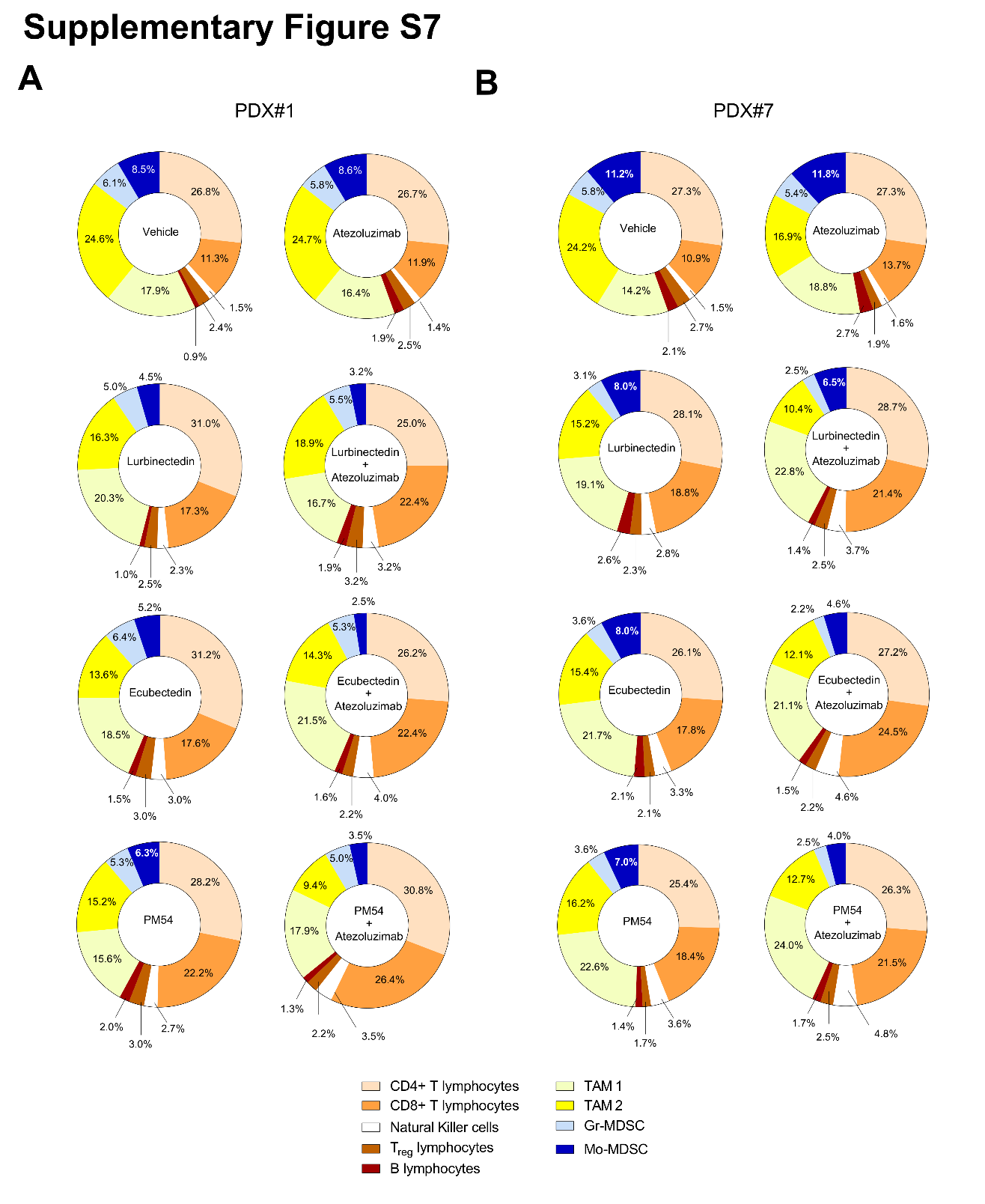


**Supplementary Figure S7. Tumor-infiltrating immune-populations infiltrating MPM unaffected by lurbinectedin, ecubectedin and PM54.** MPM#1 (epithelioid, BAP1 positive; panel A) and MPM#7 (sarcomatoid, BAP1 negative, panel B) cells were implanted s.c. in 6 week-old female Hu-CD34^+^ mice, and treated as reported under Materials and Methods. Percentage of CD4^+^T-lymphocytes, CD8^+^T-lymphocytes, NK cells, Treg-lymphocytes, B-lymphocytes, TAM1, TAM2, Gr-MDSC and Mo-MDSC infiltrating the tumors at Day 25 was measured by flow cytometry. Data are expressed as means±SD (n=4 mice/group).

## Supplementary Table S1. MPM primary samples, clinical features and treatments

| **APN** | **Histotype** | **BAP1** | **Gender** | **Age**  **(years)** | **Asbestos exposure** | **Surgery** | **1^st^-line treatment** | **2^nd^-line treatment** | **OS (months)** |
| --- | --- | --- | --- | --- | --- | --- | --- | --- | --- |
| #1 | Epithelioid | + | M | 78 | Possible | No | Palliative | No | 6 |
| #2 | Epithelioid | - | F | 74 | Yes | No | C+P | No | 13 |
| #3 | Epithelioid | + | M | 70 | Yes | Yes | No | No | 3 |
| #4 | Epithelioid | + | M | 79 | Possible | No | C+P | G | 16 |
| #5 | Epithelioid | + | M | 68 | Yes | No | C+P | P | 9 |
| #6 | Sarcomatoid | + | M | 69 | Yes | No | C+P | T | 10 |
| #7 | Sarcomatoid | - | M | 77 | Yes | No | No | No | 3 |
| #8 | Sarcomatoid | - | M | 61 | Yes | Yes | No | No | 7 |
| #9 | Biphasic | - | M | 65 | No | No | C+P | No | 11 |
| #10 | Biphasic | - | M | 55 | Possible | No | C+P | T | 10 |
| #11 | Biphasic | + | F | 69 | Yes | Yes | C+P | G | 14 |
| #12 | Biphasic | - | F | 80 | Yes | No | C+P | T | 5 |
| APN Anonymized Patient Number.  OS, Overall Survival.  M, male; F, female.  C, carboplatin; P, pemetrexed; G, gemcitabine; T, trabectedin. | | | | | | | | | |

## Supplementary Table S2. MPM primary samples histological characterization

| **APN** | **CAL** | **PANCK** | **PODO** | **EMA** | **CEA** | **WT1** | **CK5** |
| --- | --- | --- | --- | --- | --- | --- | --- |
| #1 | POS | POS | NEG | NEG | NEG | POS | NEG |
| #2 | POS | POS | NEG | NEG | NEG | POS | POS |
| #3 | NEG | POS | NEG | NEG | NEG | POS | NEG |
| #4 | POS | POS | NEG | NEG | NEG | NEG | POS |
| #5 | POS | POS | NEG | NEG | NEG | POS | POS |
| #6 | NEG | POS | NEG | NEG | NEG | NEG | NEG |
| #7 | POS | POS | NEG | NEG | NEG | FOC | NEG |
| #8 | FOC | FOC | NEG | NEG | NEG | FOC | NEG |
| #9 | POS | POS | NEG | NEG | NEG | POS | NEG |
| #10 | POS | POS | NEG | NEG | NEG | NEG | NEG |
| #11 | POS | POS | NEG | NEG | NEG | POS | NEG |
| #12 | POS | POS | NEG | NEG | NEG | POS | POS |

APN Anonymized Patient Number.

CAL: calretinin; PANCK: pancytokeratin; PODO: podoplanin; EMA: epithelial membrane antigen; CEA: carcino-embryonic antigen; WT1: Wilms tumor-1 antigen; CK5: cytokeratin 5; POS: positive; NEG: negative; FOC: focal spor: sporadic; N: nuclear

**Supplementary Table S3.** **IC_50_ (nM) of cisplatin plus pemetrexed, lurbinectedin,** **ecubectedin and PM54 in a panel of 12 patient-derived MPM cells.**

| **APN** | **Histotype** | **BAP1** | **Pt+PMX** | **lurbinectedin** | **ecubectedin** | | **PM54** | |  |
| --- | --- | --- | --- | --- | --- | --- | --- | --- | --- |
| #1 | Epithelioid | + | 1.7±0.5 | 0.6±0.2 | | 0.14±0.1 | | 0.4±0.3 | |
| #2 | Epithelioid | - | 2.9±0.7 | 0.07±0.03 | | 0.03±0.02 | | 0.02±0.007 | |
| #3 | Epithelioid | + | 1.2±0.5 | 0.06±0.02 | | 0.02±0.01 | | 0.01±0.003 | |
| #4 | Epithelioid | + | 5.6±0.4 | 0.05±0.001 | | 0.03±0.02 | | 0.02±0.003 | |
| #5 | Epithelioid | + | 4.7±0.8 | 0.06±0.002 | | 0.2±0.1 | | 0.04±0.03 | |
| #6 | Sarcomatoid | + | 4.4±0.6 | 0.5±0.08 | | 0.63±0.06 | | 0.54±0.08 | |
| #7 | Sarcomatoid | - | 9.8±1.4 | 0.11±0.008 | | 0.15±0.03 | | 0.21±0.04 | |
| #8 | Sarcomatoid | - | 6.4±1.1 | 0.09±0.04 | | 0.15±0.03 | | 0.24±0.07 | |
| #9 | Biphasic | - | 6.7±1.4 | 0.12±0.05 | | 0.11±0.04 | | 0.13±0.03 | |
| #10 | Biphasic | - | 9.4±1.1 | 0.85±0.12 | | 1.1±0.7 | | 0.23±0.04 | |
| #11 | Biphasic | + | 10.3±1.7 | 0.11±0.4 | | 0.18±0.07 | | 0.11±0.03 | |
| #12 | Biphasic | - | 8.7±1.5 | 0.3±0.07 | | 0.4±0.1 | | 0.34±0.08 | |
| APN, anonymized patient number: Pt+PMX: cisplatin+pemetrexed.  Values represent mean±SD of three independent experiments. | | | | | | | | |  |

## Supplementary Table S4. Immunophenotype of PBMC after 5 day-incubation with MPM cells.

|  | | **Untreated** | **Pt+PMX** | **L** | **ecubectedin** | **PM54** |
| --- | --- | --- | --- | --- | --- | --- |
| **Lymphoid cells** | **T-helper lymphocytes (CD3+CD4+)** | 42.3±8.1 | 40.2±6.7 | 34.6±5.4 | 32.4±6.7 | 33.4±4.9 |
|  | **T-cytotoxic lymphocytes (CD3+CD8+)** | 10.3±2.3 | 11.3±3.4 | 15.6±4.1 | 16.7±4.5 | 18.7±7.1 |
|  | **NK (CD56+CD335+)** | 2.6±0.8 | 2.1±1.1 | 3.5±1.3 | 4.2±1.2 | 5.1±0.8 ^a ,d^ |
|  | **Treg (CD4+CD25+CD127low)** | 5.4±1.2 | 4.5±1.5 | 5.2±1.4 | 3.8±1.1 | 3.1±0.6 ^a^ |
| **Myeloid cells** | **Monocytes (CD14+)** | 27.5±2.9 | 25.3±5.8 | 18.7±5.6 | 16.3±4.1 | 18.9±8.4 |
|  | **Macrophages (CD14+CD68+)** | 32.3±6.7 | 30.5±4.5 | 27.4±5.7 | 26.7±5.9 | 24.0 ±54.9 |
|  | **Gr-MDSC (CD11b+CD14+CD15+HLA-DR-cells)** | 4.2±1.4 | 2.3±1.5 | 2.1±0.8 | 2.6±1.4 | 2.5±1.5 |
|  | **Mo-MDSC (CD11b+CD14+CD15lowHLA-DR-cells)** | 13.4±4.5 | 10.4±4.5 | 7.5±2.5 | 7.2±4.2 | 6.4±2.6 ^a^ |
| PBMC from healthy volunteers were co-incubated 5 days with MPM cells, previously grown 24 h in drug-free medium (untreated), cisplatin+pemetrexed (Pt+PMX), lurbinectedin (L), ecubectedin and PM54 at their IC_50_. Then, PBMC were collected and immunophenotyped by flow cytometry. Data are expressed as means±SD of 12 MPM samples (n=3 independent experiments, in duplicates). ^a^ p<0.05: vs untreated cells; ^d^ p<0.05: vs Pt+PMX. | | | | | | |

## Supplementary Table S5. ICP/ICP ligands and immune-senescence expression on T-lymphocytes and MPM cells, after lurbinectedin, ecubectedin and PM54.

|  |  | **Untreated** | **Pt+PMX** | **L** | | **ecubectedin** | **PM54** |
| --- | --- | --- | --- | --- | --- | --- | --- |
| **MPM** | **PD-L1** | 14.5±3.5 | 10.9±3.4 | 7.8±3.1 ^a^ | | 7.3±2.5 ^a^ | 7.6±1.3 ^a^ |
|  | **PD-L2** | 8.9±3.2 | 7.5±1.7 | 8.4±1.5 | | 7.6±1.9 | 8.1±1.8 |
|  | **TIM-3** | 7.8±2.1 | 6.9±1.4 | 7.3±0.8 | | 5.2±1.5 | 5.1±1.9 |
|  | **LAG-3** | 10.3±2.4 | 9.1±1.8 | 6.3±1.4 ^a^ | | 5.3±1.9 ^a^ | 5.8±1.8 ^a^ |
|  |  |  |  |  | |  |  |
| **T-helper lymphocytes** | **PD-1** | 2.4±1.5 | 3.4±0.9 | 2.7±1.7 | | 1.7±1.1 | 2.4±0.7 |
|  | **TIM-3** | 5.4±2.0 | 4.9±3.4 | 3.4±1.1 | | 3.1±1.2 | 3.4±1.3 |
|  | **LAG-3** | 9.8±3.1 | 7.9±3.2 | 5.6±2.3 | | 4.1±1.9 ^a^ | 4.4±0.7 ^a^ |
|  | **CTLA-4** | 6.7±3.5 | 6.1±3.1 | 7.2±4.3 | | 6.4±3.2 | 6.1±1.9 |
|  | **HVEM** | 3.4±2.5 | 2.9±1.2 | 2.8±1.2 | | 3.4±3.2 | 2.1±1.1 |
|  | **TIGIT** | 3.9±1.2 | 2.5±1.1 | 4.1±0.8 | | 3.9±2.1 | 3.2±1.1 |
|  | **CD160** | 4.8±2.4 | 4.5±2.1 | 2.4±1.6 | | 2.8±1.2 | 2.9±1.7 |
|  | **CD57** | 12.7±3.6 | 10.8±3.1 | 7.2±2.1 | | 5.9±1.7 ^a, d^ | 4.3±1.2 ^a, d^ |
|  |  |  |  |  | |  |  |
| **T-cytotoxic lymphocyt**es | **PD-1** | 43.7±10.9 | 34.5±9.1 | 27.8±8.3 ^a^ | | 23.4±9.1 ^a^ | 28.9±7.3 ^a^ |
|  | **TIM-3** | 5.4±1.6 | 4.6±1.3 | 5.1±1.5 | | 4.9±1.4 | 4.8±1.1 |
|  | **LAG-3** | 9.8±1.8 | 7.8±1.9 | 5.6±1.4 ^a^ | | 5.1±1.4 ^a^ | 4.9 ± 0.8 ^a^ |
|  | **CTLA-4** | 4.3±1.3 | 4.3±2.1 | 5.4±1.4 | | 4.7±1.4 | 4.6 ± 1.2 |
|  | **HVEM** | 2.1±1.8 | 2.8±1.4 | 2.4±1.2 | | 2.6±1.1 | 2.6 ± 0.8 |
|  | **TIGIT** | 6.7±1.9 | 6.6±1.1 | 5.8±1.6 | | 5.3±1.2 | 5.9 ± 0.8 |
|  | **CD160** | 7.6±2.1 | 6.5±2.1 | 4.9±1.8 | | 5.5±1.2 | 5.6 ± 1.3 |
|  | **CD57** | 39.9±7.8 | 32.9±5.9 | 21.4±3.8 ^a, d^ | | 19.8±4.9 ^a, d^ | 21.3 ± 6.3 ^a, d^ |
|  | |  |  |  | |  |  |
| **NK** | **PD-1** | 26.3 ± 4.9 | 29.8 ± 5.6 | | 13.8 ± 2.5 ^a, d^ | 12.4 ± 2.5 ^a, d^ | 13.4 ± 3.2 ^a, d^ |
|  | **TIM-3** | 6.7 ± 2.1 | 6.5 ± 1.4 | | 5.9 ± 1.8 | 6.3 ± 1.4 | 7.5 ± 3.4 |
|  | **LAG-3** | 4.9 ± 1.1 | 4.7 ± 1.1 | | 5.9 ± 1.4 | 4.8 ± 1.7 | 5.4 ± 1.3 |
|  | **CTLA-4** | 4.5 ± 1.2 | 5.6 ± 1.2 | | 5.8 ± 1.5 | 5.3 ± 1.4 | 5.2 ± 1.9 |
|  | **HVEM** | 4.3 ± 0.9 | 4.5 ± 2.3 | | 4.9 ± 1.8 | 4.7 ± 1.1 | 4.3 ± 1.3 |
|  | **TIGIT** | 5.6 ± 1.2 | 4.3 ± 1.1 | | 5.6 ± 1.4 | 5.6 ± 1.7 | 6.3 ± 1.1 |
|  | **CD160** | 9.8 ± 1.3 | 8.5 ± 1.8 | | 7.6 ± 2.1 | 8.5 ± 1.4 | 8.9 ± 1.9 |
|  | **CD57** | 25.7 ± 2.4 | 19.8 ± 4.5 | | 17.8 ± 3.4 ^a^ | 14.3 ± 2.8 ^a^ | 11.4 ± 2.9 ^a^ |
| MPM cells were grown 24 h in drug-free medium (untreated), cisplatin+pemetrexed (Pt+PMX), lurbinectedin (L), ecubectedin and PM54 at their IC_50_. Then, an aliquot was used to quantify the expression of ICP ligands by flow cytometry. A second aliquot was washed and incubated 5 days with the PBMC of healthy donors. After this, ICP and immune-senescence markers were evaluated by flow cytometry on isolated CD4^+^T-helper lymphocytes, CD8^+^T-cytotoxic lymphocytes and NK cells. Data are expressed as means±SD of 12 MPM samples (n=3 independent experiments, in duplicates). ^a^ p<0.05: vs untreated cells; ^d^ p<0.05: vs Pt+PMX. | | | | | | | |

## Supplementary Table S6. Quantification of the immune-infiltrating cells in excised MPM1 and MPM7 implanted in Hu-NSG mice.

| **MPM#1** |  | **vehicle** | **Pt+PMX** | **L** | **A** | **L+A** | **Ecu** | **Ecu+A** | **PM54** | **PM54+A** |
| --- | --- | --- | --- | --- | --- | --- | --- | --- | --- | --- |
| **Lymphoid cells** | T-helper lymphocytes (CD3+CD4+) | 43.0 ±12.3 | 51.0 ± 18.3 | 62.0 ± 13.1 | 48.2 ± 5.8 | 41.2 ± 7.0 | 52.3 ± 6.2 | 44.5±8.7 | 40.3 ± 7.9 | 49.8 ± 14.7 |
|  | T-cytotoxic lymphocytes (CD3+CD8+) | 19.0 ± 7.9 | 20.5 ± 6.9 | 33.0 ±.4 ^b^ | 22.0 ± 2.9 | 36.8±6.2 ^b, d^ | 28.8 ± 4.0 | 36.0±4.2 ^b, d^ | 34.0 ±2.9 ^b, e^ | 41.5 ±3.4 ^c, f^ |
|  | NK (CD56+CD335+) | 2.8±1.7 | 3.5 ± 1.3 | 4.5 ± 1.3 | 2.7 ± 0.9 | 5.0 ± 0.8 ^a^ | 5.0 ± 0.6 ^a^ | 6.5 ± 1.3 ^a^ | 4.5 ± 1.7 | 6.0 ± 1.4 ^a^ |
|  | Treg (CD4+CD25+CD127low) | 4.0 ± 8.0 | 4.5 ± 1.3 | 5.0 ± 1.8 | 4.7±0.9 | 4.8 ± 1.5 | 5.0±1.8 | 3.8 ± 1.0 | 4.3 ± 1 | 3.5 ± 1.3 |
| **Myeloid cells** | TAM1 (CD68+CD86+iNOS+) | 31.8 ±10.0 | 45.5 ± 2.6 | 40.3 ± 6.8 | 29.7± 6.8 | 26.3 ± 4.3 | 31.8± 8.5 | 34.0±2.2 | 26.3 ± 7.3 | 27.5 ± 5.1 |
|  | TAM2 (CD68+CD206+Arg1+) | 40.5 ± 5.4 | 30.5 ± 4.5 | 34.3 ±7.7 | 42.2± 7.1 | 30.3 ± 6.4 | 21.5±3.3 ^c, e^ | 23.8±7.2 ^b^ | 25.0±6.4 ^b^ | 15.0±1.8 ^c, f^ |
|  | Gr-MDSC (CD11b+CD14+CD15+HLA-DR-cells) | 9.8 ± 2.2 | 12.3 ± 3.1 | 9.5 ± 2.6 | 10.2 ± 3.5 | 8.3 ± 2.5 | 10.5 ± 2.1 | 8.5 ± 2.1 | 7.5 ± 2.1 | 8.0 ± 3.7 |
|  | Mo-MDSC (CD11b+CD14+CD15lowHLA-DR-cells) | 15.0 ± 5.1 | 15.8 ± 2.2 | 9.0 ± 1.8 | 15.0 ± 2.1 | 4.8±1.5 ^c, f^ | 9.3 ± 1.9 | 4.3±2.1 ^c, f^ | 9.3 ± 1.7 | 5.5±2.4 ^c, f^ |

| **MPM#7** |  | **Untreated** | **Pt+PMX** | **L** | **A** | **L+A** | **Ecu** | **Ecu+A** | **PM54** | **PM54+A** |
| --- | --- | --- | --- | --- | --- | --- | --- | --- | --- | --- |
| **Lymphoid cells** | T-helper lymphocytes (CD3+CD4+) | 47.3 ±12.9 | 47.5 ± 4.7 | 56.8 ± 7.1 | 53 + 11.5 | 50.5 ± 8.4 | 44.8 ± 3.1 | 42.8±6.8 | 44.3 ± 12.5 | 44.8 ± 16.5 |
|  | T-cytotoxic lymphocytes (CD3+CD8+) | 18.05 ± 4.8 | 24.0 ± 2.4 | 37.0±3.9 ^c, f^ | 26.2 ± 5.3 | 38.5±5.3 ^c, f^ | 29.3±4.6 ^b^ | 39.8±7.9 ^c, e^ | 33.8±3.1 ^c, e^ | 37.3±8.5 ^c, e^ |
|  | NK (CD56+CD335+) | 2.5±1.3 | 2.8 ± 1.0 | 5.0 ± 1.4 ^a^ | 3.0 ± 1.8 | 6.5 ± 1.3 ^b, d^ | 5.8 ± 1.0 ^b, d^ | 7.5±1.3 ^c, e^ | 6.0 ± 1.4 ^b, e^ | 8.0±2.2 ^c, f^ |
|  | Treg (CD4+CD25+CD127low) | 4.5 ± 1.3 | 3.3 ± 1.0 | 4.5 ± 1.3 | 3.7±1.7 | 4.3 ± 1.0 | 3.8±1.7 | 3.8 ± 1.7 | 3.8 ± 2.4 | 4.3 ± 2.8 |
| **Myeloid cells** | TAM1 (CD68+CD86+iNOS+) | 21.5 ±5.1 | 25.0±7.3 | 36.8 ± 4.0 ^a^ | 33.2±5.7 ^a^ | 39.5±4.0 ^b, d^ | 36.8±2.1 ^c, f^ | 34.5±3.0 ^c, f^ | 39.3±5.5 ^c, f^ | 42.8±5.1 ^c, f^ |
|  | TAM2 (CD68+CD206+Arg1+) | 40.3 ± 6.2 | 37.0 ± 4.7 | 29.3±3.9 ^b, d^ | 31.5± 9.4 | 18.8±5.0 ^c, f^ | 26.8 ± 3.1 ^a, d^ | 19.3±5.3^c, f^ | 29.5 ± 5.9 ^b, d^ | 21.8±5.1 ^c, f^ |
|  | Gr-MDSC (CD11b+CD14+CD15+HLA-DR-cells) | 9.0 ± 3.2 | 8.3 ± 3.3 | 6.5 ± 1.9 | 9.7 ± 3.7 | 4.8 ± 1.7 ^a, d^ | 6.0 ± 1.8 | 4.0 ± 1.4 ^a, d^ | 6.8 ± 1.7 | 4.8 ± 1.7 ^a, d^ |
|  | Mo-MDSC (CD11b+CD14+CD15lowHLA-DR-cells) | 18.5 ± 5.2 | 20.0 ± 7.1 | 14.8± 2.6 | 20.7 ± 4.8 | 12.5 ± 3.9 | 13.3 ± 1.7 | 7.8 ± 1.7 ^b, e^ | 13.5 ± 2.4 | 7.0 ± 1.8 ^b, e^ |
| MPM#1 (epithelioid, BAP1 positive) and MPM#7 (sarcomatoid, BAP1 negative) cells were implanted s.c. in 6 week-old female Hu-CD34+ mice, and treated as reported under Materials and methods. The percentage of CD4^+^T-lymphocytes, CD8^+^T-lymphocytes, NK cells, Treg cells, TAM1, TAM2, Gr-MDSC and Mo-MDSC infiltrating the tumors at Day 25 was measured by flow cytometry. Data are expressed as means±SD (n=4 mice/group). ^a^ p<0.05, ^b^ p<0.01, ^c^ p<0.001: vs untreated cells; ^d^ p<0.05, ^e^ p<0.01,^f^ p<0.001: vs Pt+PMX. | | | | | | | | | | |
